# An Investigation of the Effect of the Traditional Naxi Herbal Formula Against Liver Cancer Through Network Pharmacology, Molecular Docking, and In Vitro Experiments

**DOI:** 10.3390/ph17111429

**Published:** 2024-10-25

**Authors:** Xiuxiang Yan, Angkhana Inta, Xuefei Yang, Hataichanok Pandith, Terd Disayathanoowat, Lixin Yang

**Affiliations:** 1 Key Laboratory of Economic Plants and Biotechnology, Kunming Institute of Botany, Chinese Academy of Sciences, Kunming 650201, China; yanxiuxiang@mail.kib.ac.cn (X.Y.); xuefei@mail.kib.ac.cn (X.Y.); 2 Department of Biology, Faculty of Science, Chiang Mai University, 239 Huay Kaew Road, Chiang Mai 50200, Thailand; aungkanainta@hotmail.com (A.I.); hataichanok064@gmail.com (H.P.); 3 Yunnan International Joint Laboratory of Southeast Asia Biodiversity Conservation, Menglun 666303, China; 4 Southeast Asia Biodiversity Research Institute, Chinese Academy of Sciences, Yezin, Nay Pyi Taw 05282, Myanmar

**Keywords:** network pharmacology, molecular docking, in vitro experiments, underlying mechanism, the formula Chong-Lou-Yao-Fang (CLYF), traditional Naxi herbal formula, liver cancer

## Abstract

**Background/Objectives**: The formula Chong-Lou-Yao-Fang (CLYF) is an herbal medicinal formulation developed by the indigenous Naxi people for treating liver cancer. This study was to reveal the biological activity, potential targets, and molecular mechanisms of CLYF for cancer treatment. **Methods**: Network pharmacology, microarray data analysis, survival analysis, and molecular docking were employed to predict potential compounds, targets, and pathways for the treatment of liver cancer. In vitro experiments and Western blot validation were conducted to confirm these predictions. **Results**: 35 key compounds and 20 core targets were screened from CLYF, involving signaling pathways for PI3K–Akt, MAPK, hepatitis B and C, which were effective for liver cancer treatment. Microarray data analysis and survival analysis indicated that EGFR and TP53 serve as promising biomarkers for diagnosis and prognosis in liver cancer. Molecular docking revealed stable binding between EGFR, TP53, and AKT1 with active ingredients. Cell experiments confirmed that CLYF-A suppressed cell proliferation, induced apoptosis, and caused cell cycle arrest in HepG2 cells, which were associated with a loss of mitochondrial membrane potential. Compared to the control group, the relative protein expression levels of EGFR and AKT1 significantly decreased following treatment with CLYF-A, while TP53 levels increased significantly. **Conclusions**: Verification of the anticancer activity of CLYF and its potential mechanisms may have important implications for anticancer therapies. Our results may provide a scientific basis for the clinical use of CLYF for cancer treatment and have important implications for developing pharmaceutical preparations, which also need more pharmacological experiments, clinical experiments, and in vivo experiments.

## 1. Introduction

Cancer is the leading cause of human deaths worldwide. Moreover, cancer ranks as the first or second leading cause of death before the age of 70 in 112 out of 183 countries and ranks third or fourth in another 23 countries according to estimates from the World Health Organization (WHO) for 2019 [1,2]. Hepatocellular carcinoma (HCC) is the most common type of primary liver cancer, and it is the third leading cause of cancer-related deaths worldwide [1,3,4]. Although the exact numbers change from year to year, global cancer statistics for 2020 still rank liver cancer as the third highest cause of mortality (8.3%), with lung cancer (18%) and colorectal cancer (9.4%) being the first and second most common types of cancer for that year, respectively [1,5]. At present, there are many ways to treat liver cancer, including surgery, chemotherapy, radiotherapy, targeted therapy, and immunotherapy [6,7]. However, the use of chemotherapy and radiotherapy is leading to an increase in the prevalence of drug resistance in cancer. Moreover, immunotherapy is a limited treatment option that is only applicable to some patients. Moreover, chemotherapy and radiotherapy often produce adverse effects, such as mucositis, neurotoxicity, and extravasation [7,8]. Therefore, further research is needed to develop effective cancer drugs with low toxicity and high efficiency. Traditional Chinese medicine (TCM) is frequently employed as an adjunct therapy to alleviate the adverse reactions associated with radiotherapy and chemotherapy [7,9,10,11,12]. The natural products found in TCM can enhance the therapeutic effects of chemotherapy, thereby improving patients’ quality of life and prolonging survival [13,14,15].

The balance-regulation theory is used to regulate the integrity of the human body in TCM [16]. Specifically, traditional Chinese herbal formulas (Fang-ji, Yao-fang, or Fu-fang in Chinese) composed of several herbs are considered a cultural treasure and have been passed down from generation to generation and used in China for thousands of years [17]. Many formulas have been reported to have effective anti-liver and anti-lung cancer effects, such as “Fei Yan Ning”, traditionally used as an adjuvant therapy for lung cancer [7]. Another preparation named “Yangzheng Xiaoji Jiaonang” is also used to treat liver cancer [18]. Naxi medicine is one kind of TCM, which was widely used by Naxi people in Northwestern Yunnan, China. However, in many cases, Naxi medicine is prescribed without a clear understanding of the underlying mechanism. The Chong-Lou-Yao-Fang (CLYF) herbal medicinal formulation is documented in traditional Naxi medicine, with its composition passed down through generations. For hundreds of years, CLYF has been effectively used to treat diseases and symptoms associated with liver cancer in the Northwestern Yunnan, China. CLYF comprises 11 herbs (Table S1), *Paris polyphylla* var. *yunnanensis* (Franch.) Hand.-Mazz. (CL), *Panax bipinnatifidus* Seem. (ZZS), *Panax notoginseng* (Burkill) F. H. Chen ex C. H. Chow (SQ), *Fritillaria cirrhosa* D. Don (CBM), *Pleione bulbocodioides* (Franch.) Rolfe (DSL), *Psammosilene tunicoides* W. C. Wu & C. Y. Wu (JTS), *Panax quinquefolius* L. (XYS), *Engleromyces sinensis* M.A. Whalley, Khalil, T.Z. Wei, Y.J. Yao & Whalley (ZJ), *Cynanchum otophyllum* C. K. Schneid. (QYS), *Glycyrrhiza yunnanensis* S. H. Cheng & L. K. Dai ex P. C. Li (GC), and *Gastrodia elata* Bl. (TM). CL is an herb with potential anti-cancer properties, as demonstrated in our previous research [12,19,20].

Chinese medicine formulas comprise multiple herbs, each containing active ingredients with various potential targets. This complexity makes it challenging to study and fully understand the pharmacological basis of their anti-cancer properties. The concept of “network pharmacology” was first proposed by the British pharmacologist A. L. Hopkins [21,22], based on which a four-tiered interaction network, “drug-component-target-disease”, was constructed to predict the mechanism of TCM and its formulas [23,24,25,26,27,28,29,30]. Network pharmacology has been instrumental in elucidating the key compounds present in the formula Le-Cao-Shi (LCS), which is active against hepatitis B, as well as the target proteins involved [16]. The main active compounds in the leaves of *Eucommia ulmoides* Oliver were predicted through network pharmacology for renal protection [24]. Molecular docking is used to evaluate the likelihood of an interaction between a compound and its putative target protein in the human body, which involves calculating the docking energy of the compound and predicting its binding form within its target [31,32]. The interaction between the candidate compounds in LCS and the target protein in the human body was verified through molecular docking [16]. However, the underlying mechanisms of studying Naxi medicine through network pharmacology and molecular docking have not yet been reported.

**Figure 1:**
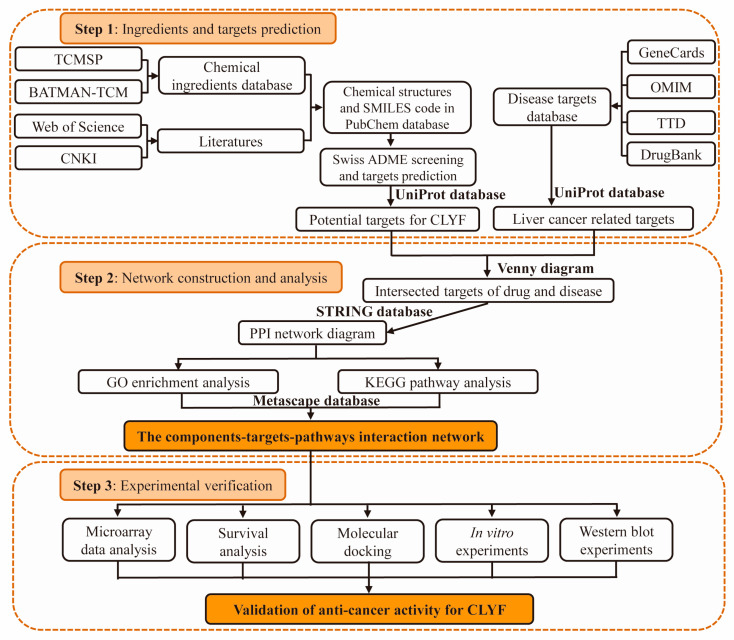
Analysis workflow of network pharmacology.

In this study, a network pharmacology strategy was used to uncover the active compounds and potential targets of the traditional Naxi CLYF formula for the treatment of liver cancer. Enrichment analyses of these putative targets were conducted for Gene Ontology (GO) terms and Kyoto Encyclopedia of Genes and Genomes (KEGG) pathways, which were conducted to explore the potential mechanisms by which the CLYF formula exerts its anti-cancer effects. The interaction between the main compounds of the CLYF formula and their key putative therapeutic targets was validated through molecular docking simulations, combined with microarray data analysis and survival analysis. These interactions were further confirmed in vitro using a liver cancer cell line, providing validation for the network pharmacological approach. The potential anti-cancer mechanism of the CLYF formula was further verified through Western blot experiments. In summary, the purpose of this study was to validate the anti-cancer activity for the pro-apoptotic and cytotoxic effects of the traditional Naxi formula CLYF. This research provides a scientific basis for the application and optimization of the formula. A specific flow chart (Figure 1) is used to achieve the above objectives.

## 2. Results

### 2.1. Screening for the Active Ingredients in CLYF and Their Therapeutic Targets

The compounds in the CLYF formulation were obtained through the Traditional Chinese Medicine Systems Pharmacology Database and Analysis Platform (TCMSP) and Bioinformatics Analysis Tool for Molecular Mechanism of Traditional Chinese Medicine (BATMAN-TCM) databases, as well as literature research. The active ingredients included 5 chemicals in *P. polyphylla* var. *yunnanensis* (CL), 3 in *P. bipinnatifidus* (ZZS), 41 in *P. notoginseng* (SQ), 19 in *F. cirrhosa* (CBM), 7 in *P. bulbocodioides* (DSL), 1 in *P. tunicoides* (JTS), 40 in *P. quinquefolius* (XYS), 12 in *E. sinensis* (ZJ), 9 in *C. otophyllum* (QYS), 39 in *G. yunnanensis* (GC), and 14 in *G. elata* (TM). Eight compounds were found in two herbs, and another three compounds were found in three herbs. A total of 176 chemical compounds and 1130 related targets were found (Table S2). The Cytoscape 3.7.1 software was employed to visualize an herb–compound–putative target network diagram, which comprised 1314 nodes and 4284 edges (Figure S1). Multiple compounds could be linked to a shared target, while conversely, multiple targets may be associated with a unique compound in one or more herbs. For instance, the herb JTS only contains one active compound, psammosilenin A, which is associated with 41 putative targets. These results suggest that a single compound may exert anti-cancer effects through multiple targets or that several compounds may share similar anti-cancer properties. Additionally, the three herbs (ZZS, SQ, XYS) from the same *Panax* genus may exhibit pharmacological similarities.

### 2.2. Targets for Liver Cancer

**Figure 2:**
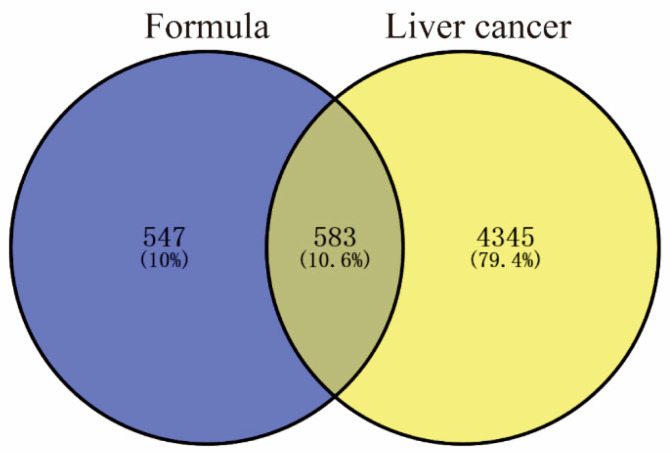
Venn diagram analysis of CLYF targets and putative therapeutic targets for the treatment of liver cancer.

Therapeutic targets were identified by searching the GeneCards, OMIM, TTD, and DrugBank databases using “Liver cancer” as the keyword. The results from all four databases were combined and filtered to create a non-redundant list, yielding a total of 4928 putative therapeutic targets. Subsequently, the union of targets related to CLYF compounds and the disease was visualized using a Venn diagram, resulting in 583 intersection targets associated with both liver cancer and the CLYF prescription (Figure 2).

### 2.3. Protein–Protein Interaction Network of the Putative Therapeutic Targets of CLYF for Treating Liver Cancer

**Figure 3:**
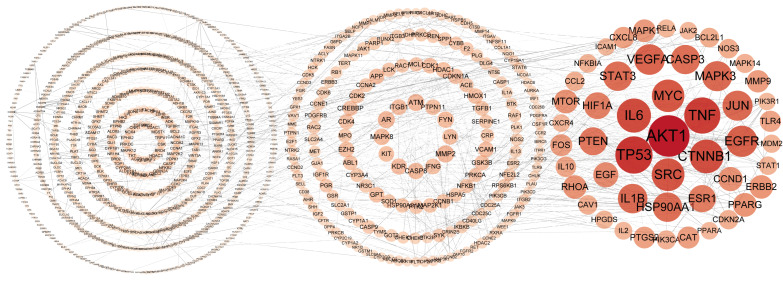
Construction of the protein–protein interaction network. The network nodes represent proteins and are represented by circles. The size and color depth of the node represent the degree value, where the larger the node and the darker the color, the larger the degree value.

The list of 583 proteins identified was submitted to the String database to obtain their putative interactors. The resulting dataset was visualized in Cytoscape 3.7.1, where the protein–protein interaction (PPI) network comprised 880 nodes and 21,023 edges (Figure 3). Putative key therapeutic targets were identified based on their degree value ranking, which represent the number of connections for each protein. The average node degree across the entire network was 47.780, with an average local clustering coefficient of 0.425, as calculated by Cytoscape 3.7.1. The top 20 identified targets were AKT1, TP53, TNF, IL6, CTNNB1, SRC, MYC, EGFR, VEGFA, JUN, MAPK3, IL1B, STAT3, HSP90AA1, CASP3, PTEN, ESR1, HIF1A, EGF, and CCND1 (Table S3, including their full names), which are primarily associated with apoptosis in liver cancer cell lines.

### 2.4. Determining the Main Active Compounds of CLYF Against Liver Cancer Through Target–Pathway Network Analysis

**Figure 4:**
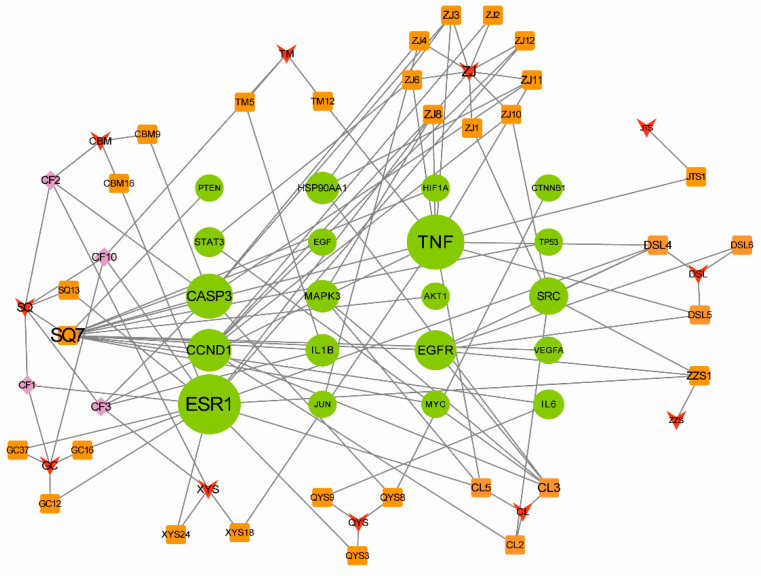
Interaction network of the main active compounds of each herb in CLYF and their key therapeutic targets. The 11 herbs comprising the CLYF formulation are shown in red; the predicted top 20 anti-cancer therapeutic targets of CLYF are shown in green; CF1, CF2, CF3, and CF10 are present in more than one of the 11 herbs constituting the CLYF formulation and are shown in purple; the top 35 compounds present in the 11 herbs are shown in orange. The size of the circles for nodes represents the degree value.

The top 20 putative therapeutic targets were selected, and then, a new network was constructed in Cytoscape 3.7.1 to illustrate the connections between these targets and their associated compounds derived from each herb in the CLYF formulation (Figure 4). The active compounds were ranked according to their degree value within this new network, resulting in a list of 35 compounds. Notably, the top active ingredients identified were SQ7 (quercetin), CL5 (20-hydroxyecdysone), CF3 (ginsenoside Rh2), DSL4 (blestriarene A), DSL5 (pleionesin C), JTS1 (psammosilenin A), XYS18 (phlegmariuine-N), ZJ3 (19,20-epoxycytochalasin D), ZJ6 (jiangxienone), ZJ8 (3,5,9-trihydroxyergosta-7,22-dien-6-one), ZJ11 (epoxycytochalasin D), TM12 (sucrose), QYS9 (arjunolic acid), QYS8 (glycosmisic acid), CL2 (diosgenin), ZZS1 (4′-hydroxywogonin), ZJ1 (cytochalasin D), ZJ10 (cytochalasin C), CL3 (pennogenin), DSL6 (shanciol H), ZJ4 (trichothecin), TM5 (M-hydroxybenzoic acid), CF2 (beta-sitosterol), CBM9 (verticinone), CF1 (liquiritigenin), SQ13 (sandaracopimarinol), CF10 (gamma-sitosterol), CBM16 (solanidine), XYS24 (pulegone), ZJ12 (cerevisterol), QYS3 (Baishouwubenzophenone), GC12 (2-Methyl-1,3,6-trihydroxyanthraquinone), GC16 (glycyrrhetol), GC37 (3-methyl-6,7,8-trihydropyrrolo[1,2-A]pyrimidin-2-one), and ZJ2 (rosenonolactone) (Table S4). As a result, these active compounds are the key ingredients in CLYF, showing potential for the treatment of liver cancer.

### 2.5. GO and KEGG Enrichment Analyses Identifies Key Signaling Pathways in the Treatment of Liver Cancer Using CLYF

**Figure 5:**
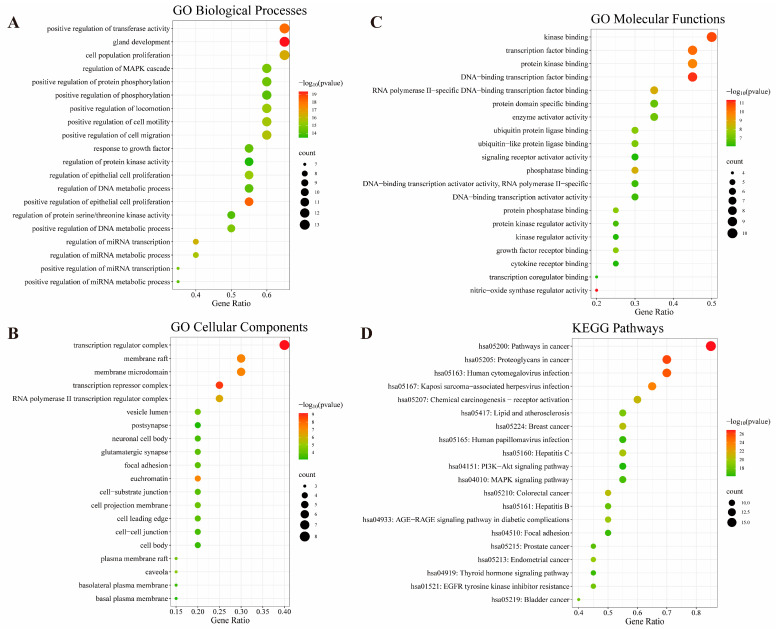
GO and KEGG pathway enrichment analyses of the top predicted therapeutic targets of CLYF. (**A**) Top 20 terms for the GO biological process (BP) category among CLYF therapeutic targets. (**B**) Top 20 terms for the GO cellular component (CC) category among CLYF therapeutic targets. (**C**) Top 20 terms for the GO molecular function (MF) category among CLYF therapeutic targets. (**D**) Top 20 enriched KEGG pathways among CLYF therapeutic targets.

GO and KEGG pathway enrichment analyses were performed using the top 20 putative therapeutic targets of CLYF with the Metascape database (Figure 5). This analysis identified 771 significantly enriched GO terms in the biological process (BP) category, 39 in the cellular component (CC) category, and 45 in the molecular function (MF) category were identified. Additionally, KEGG enrichment analysis revealed 128 significantly enriched pathways. We extracted the top 20 terms for each of the BP, CC, and MF categories (Figure 5, Table 1).

The top BP terms primarily included “positive regulation of epithelial cell proliferation”, “positive regulation of transferase activity”, and “gland development”. These terms are closely related to the regulation of apoptosis, proliferation, phosphorylation, enzyme activity, DNA, microRNA metabolism, and transcription (Figure 5A). The top CC terms encompassed “transcription regulator complex”, “transcription repressor complex”, “membrane raft”, “membrane microdomain”, and “euchromatin” (Figure 5B). The top MF terms were mainly associated with “DNA-binding transcription factor binding”, “kinase binding”, “transcription factor binding”, and “nitric oxide synthase regulator activity” (Figure 5C).

According to the KEGG pathway enrichment analysis, the top 20 putative therapeutic targets of CLYF were associated with four main signaling pathways: the “Phosphatidylinositol-3-kinase (PI3K)-Protein Kinase B (Akt)” (PI3K–Akt), “mitogen-activated protein kinase” (MAPK), “thyroid hormone”, and the “Advanced glycation end products (AGE)−Receptor of AGE (RAGE) signaling pathway in diabetic complications”. Additionally, six KEGG pathways were linked to cancer, including “cancer”, “breast cancer”, “colorectal cancer”, “prostate cancer”, “endometrial cancer”, and “bladder cancer”. Three other KEGG pathways were associated with viral infections: “human cytomegalovirus infection”, “kaposi sarcoma-associated herpesvirus infection”, and “human papillomavirus infection”. Furthermore, the KEGG pathway “EGFR tyrosine kinase inhibitor resistance” was also identified among the significantly enriched pathways. Notably, KEGG pathway enrichment analysis indicated an association between putative therapeutic targets related to CLYF and hepatitis B and C (Figure 5D, Table 1). Based on these findings, the anti-cancer properties of CLYF may involve interactions with multiple pathways, multiple targets, multiple biological processes, multiple molecular functions, and cell components.

### 2.6. Network Pharmacology Analysis Through the Generation of a Compound–Therapeutic Target–Pathway Interaction Network

**Figure 6:**
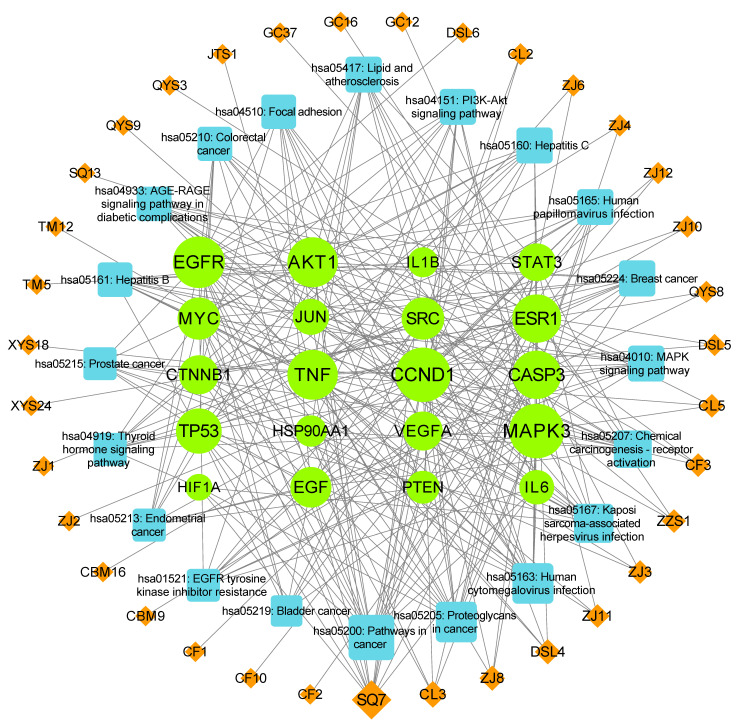
The compound–therapeutic target–pathway interaction network of the CLYF formulation. The green nodes represent the top 20 therapeutic targets; the blue nodes represent the pathways related to these therapeutic targets; the orange nodes represent the active compounds related to each therapeutic target.

To visualize the interactions among the active compounds, their putative therapeutic target proteins, and associated pathways, a compound–target–pathway network was constructed in Cytoscape 3.7.1. This network included the top 20 putative therapeutic targets, the top 20 associated KEGG pathways, and the top 35 active compounds from the CLYF formulation (Figure 6). This resulting network comprised 75 nodes and 294 edges. An additional target–pathway network was presented in Figure S2. The analysis revealed complex interactions among the various compounds, therapeutic targets, and pathways. Notably, clear visual examples were observed for the following relationships: one target leading to multiple pathways, one pathway connecting with multiple targets, one target interacting with multiple compounds, and one compound influencing multiple targets (Figure 6).

Following a network topology analysis, the degree value and betweenness centrality were employed to identify hub targets. The core therapeutic targets in this network were MAPK3 (degree = 23, betweenness centrality = 0.0621), CCND1 (degree = 23, betweenness centrality = 0.1129), EGFR (degree = 21, betweenness centrality = 0.0869), AKT1 (degree = 20, betweenness centrality = 0.0340), and TP53 (degree = 17, betweenness centrality = 0.0235). Among the 35 active compounds included in the network, the compound with the highest degree value was SQ7 (quercetin, degree = 13, betweenness centrality = 0.0378), followed by CL3 (pennogenin, degree = 5, betweenness centrality = 0.0033), ZJ8 (3,5,9-trihydroxyergosta-7,22-dien-6-one, degree = 4, betweenness centrality = 0.0151), DSL4 (blestriarene A, degree = 4, betweenness centrality = 0.0166), and ZZS1 (4′-hydroxywogonin, degree = 3, betweenness centrality = 0.0064). The core pathways related to liver cancer with the highest degree values were as follows: “hepatitis B” (degree = 10, betweenness centrality = 0.0181), “hepatitis C” (degree = 11, betweenness centrality = 0.0191), “PI3K-Akt signaling pathway” (degree = 11, betweenness centrality = 0.0116), “MAPK signaling pathway” (degree = 11, betweenness centrality = 0.0224), “thyroid hormone signaling pathway” (degree = 9, betweenness centrality = 0.0242), “EGFR tyrosine kinase inhibitor resistance” (degree = 9, betweenness centrality = 0.0089), “proteoglycans in cancer” (degree = 14, betweenness centrality = 0.0740), and “pathways in cancer” (degree = 17, betweenness centrality = 0.0865).

In the “compound–target–pathway” network, numerous instances of one pathway–multiple targets-multiple compounds were observed. Specifically, 10 potential therapeutic targets (AKT1, CASP3, IL6, JUN, MYC, MAPK3, SRC, STAT3, TNF, TP53) were directly linked to the KEGG pathway “hepatitis B” and were associated with 21 compounds (SQ7, ZJ11, ZJ3, ZJ4, CBM9, CF2, CF3, QYS8, QYS9, ZJ8, CL3, CL5, ZZS1, CL2, DSL4, ZJ10, ZJ6, DSL5, JTS1, TM12, XYS18; Table S2). Similarly, a set of 11 potential therapeutic targets (AKT1, CCND1, CASP3, CTNNB1, EGF, EGFR, MYC, MAPK3, STAT3, TNF, TP53) was directly related to the KEGG pathway “hepatitis C” and interconnected with 23 compounds (SQ7, ZJ11, ZJ3, ZJ4, CBM9, CF2, CF3, QYS8, ZJ8, CL3, CL5, ZJ6, DSL4, DSL5, JTS1, TM12, XYS18, CL2, ZJ10, ZJ12, ZJ2, DSL6, ZZS1; Table S2). Additionally, 17 potential therapeutic targets (AKT1, CCND1, CASP3, CTNNB1, EGF, EGFR, ESR1, HIF1A, HSP90AA1, IL6, JUN, MYC, MAPK3, PTEN, STAT3, TP53, VEGFA) were directly related to the KEGG pathway “cancer” and linked to 30 compounds (SQ7, ZJ11, ZJ3, ZJ4, CBM9, CF2, CF3, QYS8, QYS9, ZJ8, CL3, CL5, ZJ6, CL2, ZJ10, ZJ12, ZJ2, DSL4, DSL5, DSL6, ZZS1, CF1, CF10, GC12, GC16, GC37, QYS3, SQ13, XYS24, CBM16; Table S2). The PI3K–Akt [33,34,35,36] and MAPK [37] signaling pathways play a role in an anti-liver cancer mechanism by modulating cell cycle activity. In agreement, there were 11 potential therapeutic targets (AKT1, CCND1, EGF, EGFR, HSP90AA1, IL6, MYC, MAPK3, PTEN, TP53, VEGFA) associated with the KEGG pathway “PI3K-Akt signaling”, connected to 15 compounds (SQ7, QYS9, ZJ8, CL3, CL5, CL2, ZJ10, ZJ12, ZJ2, DSL4, DSL5, DSL6, ZZS1, ZJ11, ZJ3; Table S2). Likewise, the MAPK signaling pathway was linked to 11 potential therapeutic targets (AKT1, CASP3, EGF, EGFR, IL1B, JUN, MYC, MAPK3, TNF, TP53, VEGFA), associated with 20 compounds (SQ7, ZJ11, ZJ3, ZJ4, CBM9, CF2, CF3, QYS8, ZJ8, CL3, CL5, DSL4, DSL5, JTS1, TM12, XYS18, ZJ6, DSL6, ZZS1, TM5; Table S2).

There were several instances of one target–multiple pathways–multiple compounds (Figure 6). Three therapeutic targets were selected from the top 20 core targets for further characterization through molecular docking: EGFR [38,39], TP53 [40,41], and AKT1 [16,34]. These three putative therapeutic targets demonstrated the strongest association with liver cancer treatment based on the existing literature. EGFR was connected to 15 pathways: “cancer”, “proteoglycans in cancer”, “human cytomegalovirus infection”, “chemical carcinogenesis-receptor activation”, “colorectal cancer”, “breast cancer”, “hepatitis C”, “endometrial cancer”, “EGFR tyrosine kinase inhibitor resistance”, “bladder cancer”, “prostate cancer”, “MAPK signaling pathway”, “focal adhesion”, “human papillomavirus infection”, and “PI3K-Akt signaling pathway”. Additionally, EGFR was associated with six active compounds (CL3, DSL4, DSL5, DSL6, SQ7, ZZS1; Table S2). TP53 was connected to one compound (SQ7) and 16 pathways: “cancer”, “proteoglycans in cancer,” “human cytomegalovirus infection”, “Kaposi sarcoma-associated herpesvirus infection”, “colorectal cancer”, “breast cancer”, “hepatitis C”, “endometrial cancer”, “lipid and atherosclerosis”, “bladder cancer”, “hepatitis B”, “prostate cancer”, “MAPK signaling pathway”, “thyroid hormone signaling pathway”, “human papillomavirus infection”, and “PI3K-Akt signaling pathway”. Similarly, AKT1 was associated with one compound (SQ7) and 19 pathways: “cancer”, “proteoglycans in cancer”, “human cytomegalovirus infection”, “Kaposi sarcoma-associated herpesvirus infection”, “chemical carcinogenesis-receptor activation”, “colorectal cancer”, “breast cancer”, “hepatitis C”, “AGE-RAGE signaling pathway in diabetic complications”, “endometrial cancer”, “lipid and atherosclerosis”, “EGFR tyrosine kinase inhibitor resistance”, “hepatitis B”, “prostate cancer”, “MAPK signaling pathway”, “focal adhesion”, “thyroid hormone signaling pathway”, “human papillomavirus infection”, and “PI3K-Akt signaling pathway”.

These results reflected the involvement of multiple components, multiple therapeutic targets, and multiple pathways, suggesting a complex mechanism underlying the action of the traditional Naxi CLYF formula in the treatment of liver cancer.

### 2.7. Microarray Data Analysis and Survival Analysis

**Figure 7:**
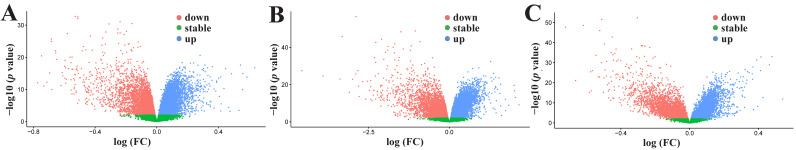
Volcano maps for microarray data analysis. (**A**) GSE136247 dataset. (**B**) GSE76427 dataset. (**C**) GSE87630 dataset. The downregulated genes are in red color, the stable genes are in green color, the upregulated genes are in blue color.

The expression differences in genes between liver cancer and normal groups were compared using the top 20 targets in three GEO databases (GSE136247, GSE76427, GSE87630). All top 20 genes were found in these three GEO databases (Figure 7). There were 10 genes (TP53, CTNNB1, SRC, MYC, VEGFA, MAPK3, HSP90AA1, CASP3, HIF1A and EGF) significantly upregulated and 10 genes (AKT1, TNF, IL6, EGFR, JUN, IL1B, STAT3, PTEN, ESR1, and CCND1) significantly downregulated in GSE136247. In GSE76427, 7 upregulated (CTNNB1, SRC, MAPK3, HSP90AA1, PTEN, HIF1A, and EGF) and 13 downregulated (AKT1, TP53, TNF, IL6, MYC, EGFR, VEGFA, JUN, IL1B, STAT3, CASP3, ESR1, and CCND1) genes were observed. In GSE87630, 9 genes (AKT1, TP53, CTNNB1, SRC, VEGFA, MAPK3, STAT3, CASP3, and EGF) were upregulated, while 11 genes (TNF, IL6, MYC, EGFR, JUN, IL1B, HSP90AA1, PTEN, ESR1, HIF1A, and CCND1) were downregulated. Both EGFR and TNF were consistently downregulated across all three GEO databases. Notably, TP53 and AKT1 were significantly downregulated in GSE76427 and upregulated in GSE87630. In GSE136247, TP53 was significantly upregulated, while AKT1 was significantly downregulated. The microarray data analysis confirmed the reliability of the screening results from network pharmacology, paving the way for further survival analysis.

**Figure 8:**
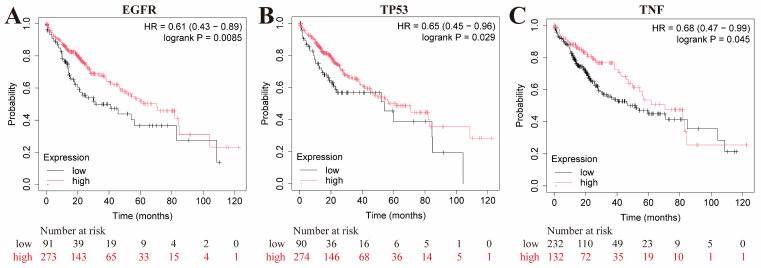
The survival analysis of core genes at *p* < 0.05. (**A**) EGFR. (**B**) TP53. (**C**) TNF. The *p* value indicates the statistical difference in the survival curve between two groups.

The Kaplan–Meier curve served as a primary representation of the survival function, illustrating the probability of an event occurring at a specific time [27]. The prognostic value of these core genes was assessed using the Kaplan–Meier survival curve (Figure 8). The results indicated that EGFR, TP53, and TNF were more relevant to the prognosis of liver cancer, suggesting that they may be considered potential targets for further research on CLYF as an anti-liver-cancer agent.

### 2.8. Molecular Docking Validation of Key Targets with Active Compounds

TCM often investigates the potential connections between or effects of certain compounds in a formula and the target proteins in the human body. The active chemicals in a drug are commonly referred to as ligands, while their target proteins can be considered receptors. Molecular docking is a technique used to evaluate the probability of an interaction between a ligand and a putative target protein, which can be quantified based on the binding energy. A negative binding energy indicates that the ligand will spontaneously bind to the target protein. If the binding energy is less than −5.0 kcal/mol, the ligand demonstrates good binding affinity to its target protein. A binding energy of less than −7.0 kcal/mol suggests an even stronger binding configuration and activity [22,42].

Generally, the CLYF formula is typically administered orally through a water decoction or powder in clinical applications. Consequently, the active ingredients in CLYF should adhere to the principles of ADME (Absorption, Distribution, Metabolism, and Excretion) screening, which further predicted the drug similarity of compounds with certain biological activity. In the process of collecting active chemical compounds in CLYF, all identified active compounds were subjected to ADME screening. According to the results of the survival analysis and literature searches [16,34,38,39,40,41], three core targets—EGFR, TP53, and AKT1—were selected for molecular docking tests, with their structures downloaded from the Protein Data Bank (PDB). All three proteins were associated with the signaling pathways related to cancer (MAPK, PI3K–Akt, and hepatitis B), while TP53 and AKT1 were also linked to the signaling pathway related to Hepatitis C (Figure 6). An SDF file for 3 out of the 35 most active compounds in CLYF could not be found (Table S4). Therefore, molecular docking tests were conducted for the 32 active ingredients in CLYF that had available SDF files, assessing their interactions with each of the three core target proteins. The binding energies are listed in Tables S5–S7, indicating that these 32 active ingredients exhibited strong binding affinities for all three core target proteins.

**Figure 9:**
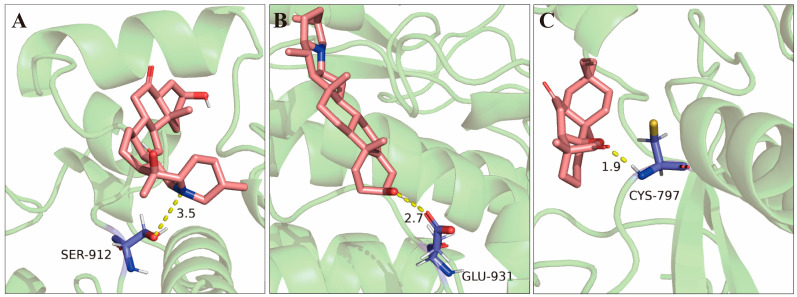
The strong binding ability between EGFR and three core active compounds of CLYF. (**A**) Molecular docking of EGFR and CBM9. (**B**) Molecular docking of EGFR and CBM16. (**C**) Molecular docking of EGFR and ZJ2.

A lower binding energy typically reflects a more stable binding conformation. For EGFR (Table S5), the five active compounds with the strongest binding ability were CBM16, followed by CL2, CL3, ZJ2, and CBM9, in decreasing order of binding energy. For TP53 (Table S6), the active compounds with the strongest binding were CBM16, CL2, GC16, CBM9, and ZJ2. In the case of AKT1, the top compounds were CBM16, ZJ2, CBM9, ZJ1, and GC16 (Table S7). Importantly, three compounds—CBM16, CBM9, and ZJ2—ranked among the top five for binding ability to all three targets. The molecular docking energies of these three compounds with the proposed therapeutic targets were summarized in Table 2. All compounds were located within the active pocket of the targets, with most forming hydrogen bonds. PyMol 2.4.0 software was employed to visualize the molecular docking of these three active compounds with the three core targets (Figure S3). Among them, EGFR exhibited the strongest binding ability with CBM9, CBM16, and ZJ2 (Table 2), with the binding energies of these three compounds with EGFR being less than −5.0 kcal/mol. According to the ADME characteristic analysis, these three compounds adhered to Lipinski rules, and their glucose absorption was predicted to be high. The numbers of rotatable bonds for these three compounds were 0, 0, and 1, respectively. The hydrogen bond acceptors for CBM9, CBM16, and ZJ2 were 4, 2, and 3, respectively, while the hydrogen bond donors were 2, 1, and 0, respectively. Therefore, the docking hydrogen bonds and their length were visualized using PyMol 2.4.0 software (Figure 9). Generally, only one hydrogen bond formed between EGFR and CBM9, named SER-912 (length of hydrogen bond = 3.5). Likewise, EGFR formed one hydrogen bond with CBM16 named GLU-931 (length of hydrogen bond = 2.7), while CYS-797 also formed hydrogen bond (length of hydrogen bond = 1.9) within the EGFR pocket (Figure 9).

### 2.9. CLYF Inhibits the Proliferation of HepG2 Cells

**Figure 10:**
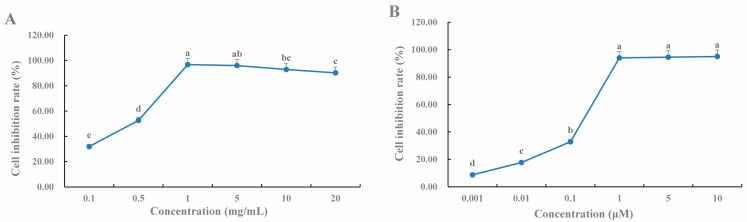
Inhibitory effect of CLYF-A extracts (**A**) and DOX (**B**) on the proliferation of HepG2 cells. Different lowercase letters indicate significant differences (*p* < 0.05).

The activity of CLYF extracts on cell proliferation was assessed using a cell counting kit with various concentrations of ethanolic, aqueous, and residual extracts. The results showed that the ethanol extract (CLYF-A) significantly inhibited the growth of tumor cells in a dose-dependent manner (Figure 10). Notably, 0.1 μM doxorubicin (DOX) is equivalent to 0.058 μg/mL, suggesting that CLYF-A was not more effective than the positive control chemotherapeutic agent DOX. The IC_50_ (half maximal inhibitory concentration) value for the CLYF-A extract was determined to be 0.2509 mg/mL (Table 3). In contrast, the aqueous extract (CLYF-W) had no activity toward cell proliferation. These findings suggest that CLYF suppresses the proliferation of liver cancer cells by inhibiting the MAPK and PI3K–Akt signaling pathways.

**Figure 11:**
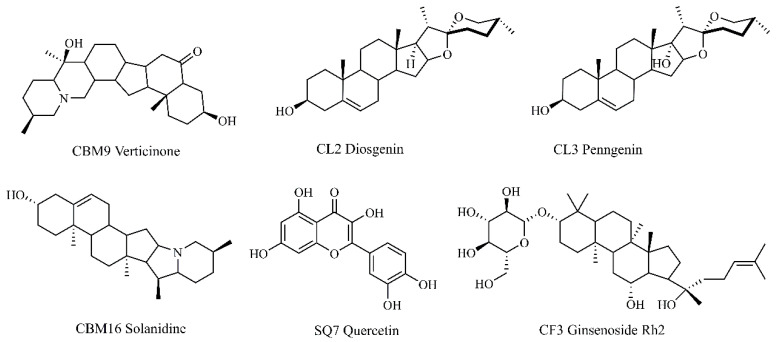
Structures of six core active compounds in CLYF-A.

CLYF can be administered in traditional Naxi medicine as a water decoction (equivalent to CLYF-W) or as a powder (equivalent to CLYF-A). To ensure consistency in the effective ingredients between the traditional edible method and modern experimental extraction method, CLYF-W was designated as the water decoction, while CLYF-A was designated as the powdered form. The results presented in Figure 10 and Table 3 show that CLYF-W showed no activity. However, CLYF-A exhibited significant inhibitory effects on HepG2 cells in a dose-dependent manner (Figure 10A). This suggested that the powdered form was more effective than water decoction. The top core active compounds, based on binding energy, all demonstrated significant inhibitory effects on the proliferation of HepG2 cell lines, also showing a clear dose-dependent response (Figure S4). Table 3 lists the IC_50_ values for the CLYF extracts and six core active compounds (Figure 11) in relation to the HepG2 cell lines.

Six core active compounds in CLYF-A were identified using HPLC (High Performance Liquid Chromatography) and compared with six standards. The percentage content of these compounds—CBM9, CBM16, SQ7, CL2, CL3, and CF3—was found to be 0.166%, 0.126%, 0.030%, 0.006%, 0.037%, and 0.005%, respectively. The results for these six hub active ingredients align with the prediction of network pharmacology.

### 2.10. Effects of CLYF on the Cell Cycle and Apoptosis

**Figure 12:**
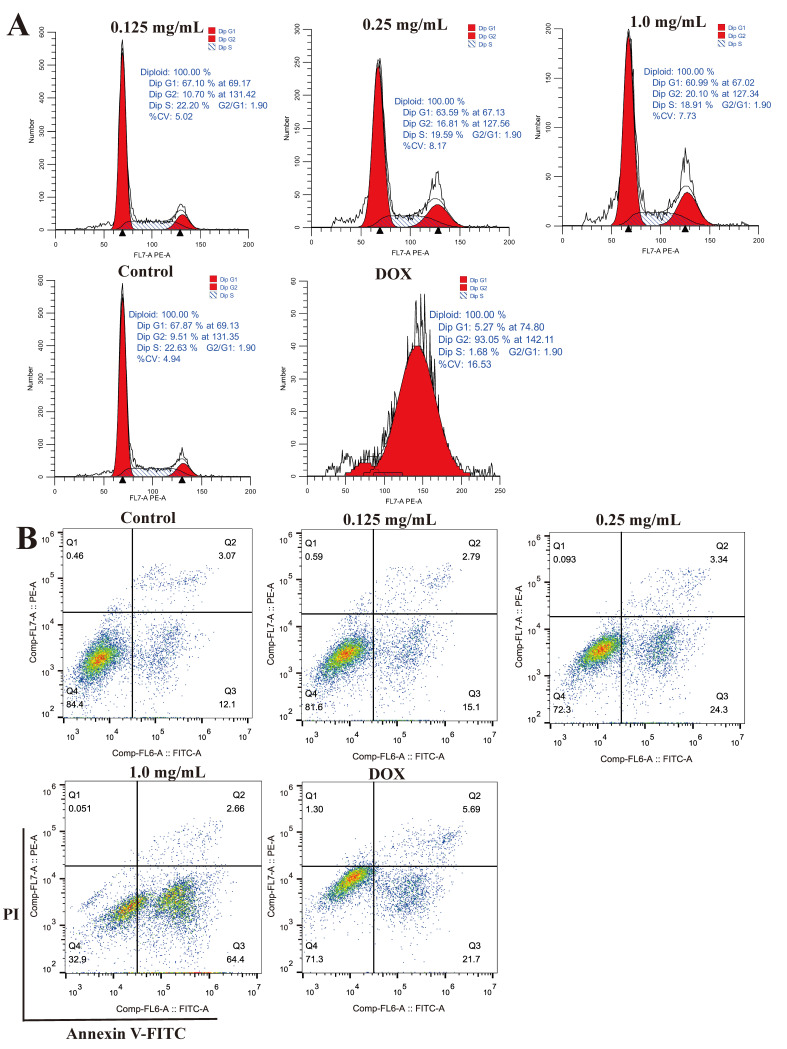
CLYF-A induces cell cycle arrest and apoptosis in HepG2 cells. HepG2 cells were treated with three concentrations (0.125, 0.25, 1.0 mg/mL) of CLYF-A. Untreated groups served as a negative control. DOX-treated groups served as a positive control at 0.5 μM. (**A**) The cell cycle analysis of HepG2 cells after treatment with CLYF-A. (**B**) CLYF-A induces apoptosis in HepG2 cells.

The results of the cell proliferation assays indicated that HepG2 cells may undergo cell cycle arrest or other forms of cell death. Flow cytometric analysis was employed to assess whether the inhibition of HepG2 cell growth affected cell cycle progression (G0/G1, S, and G2/M). Cells were treated with different concentrations of CLYF-A (0.125, 0.25, 1.0 mg/mL) along with a positive control (DOX, 0.5 μM) for 24 h. A consistent concentration of dimethyl sulfoxide (DMSO) was used as a negative control, referred to as the control group in Figure 12. Compared to the control group, treatment with CLYF-A resulted in a decrease in the number of cells in the G0/G1 phase, while the number of cells in the G2/M increased. This suggested that CLYF-A caused G2/M cell cycle arrest, similar to the effects observed in the DOX group (Figure 12A, Table 4). Double staining with annexin V–FITC and PI was performed to determine cell apoptosis. As shown in Figure 12B and Table 5, the number of cells in early-stage apoptosis (annexin V-FITC+ and PI−, Q3) increased with higher concentrations of CLYF-A treatment. Notably, the medium-dose group (0.25 mg/mL) exhibited a higher number of late-stage apoptotic cells (annexin V-FITC+ and PI+, Q2) compared to both the high-dose (1.0 mg/mL) and low-dose (0.125 mg/mL) groups. The differences among the three concentrations of CLYF-A were statistically significant when compared to the control group. As shown in Figure 12, Table 4 and Table 5, CLYF-A rapidly induced both cell cycle arrest and apoptosis in HepG2 liver cancer cell lines in a dose-dependent manner.

### 2.11. Effects of CLYF on Mitochondrial Membrane Potential

**Figure 13:**
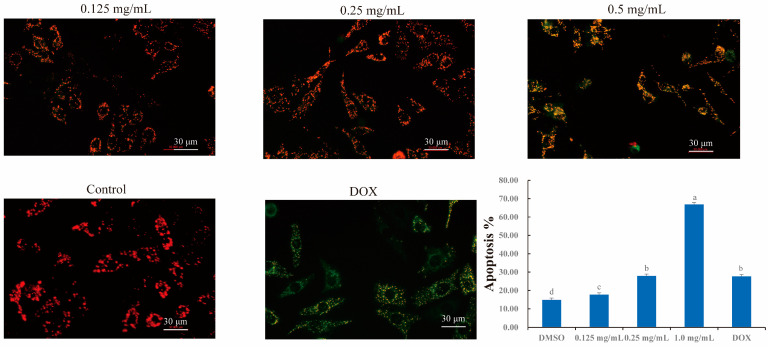
Mitochondrial membrane potential of HepG2 cells treated with CLYF-A. HepG2 cells were treated with three concentrations (0.125, 0.25, 0.5 mg/mL) of CLYF-A. Untreated groups served as a negative control. DOX-treated groups served as a positive control at 0.5 μM. Green fluorescence indicates a higher mitochondrial membrane potential. Red fluorescence indicates a lower mitochondrial membrane potential. Different lowercase letters (a, b, c, and d) indicate significant differences (*p* < 0.05).

The red fluorescence emitted by the JC-1 probe indicates a normal mitochondrial membrane potential, while a shift to green fluorescence signifies a decrease in membrane potential, indicating that the cell is in the early stages of apoptosis. The control group exhibited red fluorescence (Figure 13), serving as a negative control and indicating that the mitochondrial membrane potential was normal. In contrast, treatment with 0.5 μM DOX, used as a positive control, resulted in strong green or yellow-green fluorescence, indicating a decrease in mitochondrial membrane potential and that the cells were in the early stage of apoptosis. When compared to the negative and positive control groups, the treatment of HepG2 cells with CLYF-A induced significant and dose-dependent decreases in the mitochondrial membrane potential (Figure 13).

### 2.12. Effects of CLYF on Expression of the Core Proteins EGFR, TP53, and AKT1

**Figure 14:**
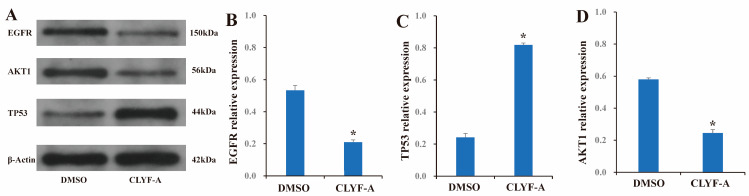
The expression of the core targets EGFR, TP53, and AKT1 in HepG2 cells after treatment with CLYF-A (0.25 mg/mL). Untreated groups served as a negative control (DMSO). (**A**) Electrophoresis graphs. (**B**) EGFR relative expression. (**C**) TP53 relative expression. (**D**) AKT1 relative expression. “*” indicates a significant difference between the CLYF-A group and the control group (*p* < 0.05).

Western blot analysis was conducted to verify the effectiveness of CLYF-A treatment in HepG2 cells based on the core proteins EGFR, TP53, and AKT1. β-Actin served as an internal reference gene, while the untreated group (DMSO) acted as a negative control. Compared to the control group, the relative protein expression levels of EGFR (Figure 14A,B) and AKT1 (Figure 14A,D) were significantly decreased following CLYF-A treatment in HepG2 cells. In contrast, the relative protein expression levels of TP53 (Figure 14A,C) were significantly increased after the treatment with CLYF-A in HepG2 cells in comparison with the control group. However, the difference (*p* < 0.05) between the CLYF-A group and the control group was statistically significant (Figure 14 and Figure S5 and Table S8).

## 3. Discussion

Naxi herbal formulas have unique advantages and potential for the treatment of complex diseases, primarily due to their multi-component and multi-target characteristics. However, the complexity of the Naxi herbal formula also poses challenges for its application and development. The Naxi formula CLYF is guided by the compatibility principles of properties, flavors, and efficacy. In the CLYF formula, the herbs XYS, GC, and QYS are utilized to invigorate qi and stimulate the spleen, while an additional five herbs are used to clear away heat and detoxify the body. The herbs CL, ZZS, SQ, CBM, DSL, JTS, and TM contribute to reducing swelling, dissipating blood stasis, relaxing the meridian, and clearing collaterals. Modern pharmacological studies have confirmed that CLYF consists of 11 herbs, with 10 exhibiting anti-tumor effects, while SQ shows no significant effect [12,43,44,45,46,47,48,49,50,51]. CL has demonstrated a range of pharmacological activities, including anti-tumor, hemostatic, anti-inflammatory, analgesic, and anti-fungal properties, as documented in indigenous books and phytochemical and pharmacological studies [12,18,52]. Among the 11 herbs, both ZZS [18,46,53,54,55] and DSL [51,56,57,58] have been reported to possess liver-protective effects, as well as the pharmacological effects of anti-cancer, anti-inflammatory, neuroprotective, and anti-oxidant effects. CBM is an important Chinese herbal medicine known for relieving cough, alleviating asthma, expelling phlegm, and providing anti-inflammation, blood pressure-lowering, anti-tumor, blood pressure-lowering, neuroprotection, analgesia, and anti-oxidation effects [45,59]. Furthermore, JTS has demonstrated anti-tumor, anti-oxidant, analgesia, and anti-rheumatoid arthritis activities in ancient indigenous books and Chinese literature [18,50,60,61]. XYS exhibits a wide range of pharmacological activities, including anti-tumor, anti-oxidative, anti-diabetic, anti-stress, anti-aging, anti-fatigue, anxiolytic anti-inflammatory, neuroprotective, and immunomodulatory effects [18,48,62,63,64,65]. It is utilized for the treatment of conditions, such as antibiotics, inflammation, and rheumatoid arthritis [32,40]. Phytochemical research on ZJ indicated that crude extracts and compounds in ZJ demonstrate anti-tumor, anti-oxidant, and anti-inflammatory effects [49,66]. Traditionally, ZJ has been used for its antibiotic, anti-inflammatory, and rheumatoid arthritis effects in indigenous books [60,67]. QYS is recognized for its pharmacological effects, including anti-cancer, anti-inflammatory, anti-hepatitis, anti-viral, neuroprotective, immunomodulatory, and activities against epilepsy and rheumatism [43,60,68,69,70]. Both licorice [44,71,72,73] and TM [18,47,73,74,75] have been reported for their pharmacological activities, including anti-tumor, antivirus, and anti-inflammatory. However, the anti-cancer properties of GC are documented in local books [60]. Overall, while CLYF has demonstrated anti-tumor efficacy, its underlying mechanisms are complex, involving multiple components and targets.

Network pharmacology analyzes the effects of drugs at a systemic level, revealing the synergistic mechanisms by which they act on multiple components, targets, and pathways within the human body. This approach aligns with the holistic perspective of TCM theory. Additionally, it offers new methodological support for the transition of the Naxi formula from traditional practices to a more theoretical scientific framework [21,22,23,24,25]. In this study, we used network pharmacology to construct the herbs–compounds–targets network, systematically revealing the substance basis and underlying mechanisms of CLYF for the treatment of liver cancer.

Network pharmacology revealed 176 active ingredients in the CLYF formulation and 583 potential therapeutic targets for liver cancer treatment. From these targets associated with the herb–disease–target intersection, the top 20 targets were selected to construct a PPI network, followed by GO and KEGG enrichment analyses. Based on the interaction information for “compound-target” in the herb–compound–putative target network diagram (Figure S1) and degree values, we found that 1 compound interacted with AKT1, TP53, CTNNB1, MYC, VEGFA, JUN, PTEN, HIF1A, and EGF; 2 compounds interacted with IL6 and STAT3; 3 compounds interacted with MAPK3, IL1B, and HSP90AA1; 5 compounds interacted with SRC; 6 compounds interacted with EGFR; 7 compounds interacted with CCND1; 8 compounds interacted with CASP3, 12 compounds interacted with TNF; and 14 compounds interacted with ESR1. After merging and deduplicating these compounds, a total of 35 compounds were found to interact with the top 20 core targets in CLYF. Active chemical compounds in CLYF were collected and screened using various ADME criteria through the TCMSP database, BATMAN-TCM database, and relevant literature. The three core targets were EGFR, TP53, and AKT1, which were further investigated through molecular docking analysis [16,34,38,39,40,41]. EGFR is a crucial regulator of angiogenesis [76], primarily involved in the maturation of neovascularization and the initiation of tumor angiogenesis, which is closely related to the proliferation of blood vessels in liver cancer [77]. Consequently, the predicted anti-tumor mechanisms of EGFR include cell cycle arrest, the promotion of apoptosis, inhibition of tumor invasion and metastasis, and anti-angiogenesis. Its activated signaling pathways can regulate tumor cell proliferation, differentiation, survival, and cell cycle progression and angiogenesis [78]. TP53 is a tumor-suppressor protein highly correlated with tumorigenesis; it regulates the cell cycle and induces cell apoptosis. While TP53 is present at low levels in normal cells, it accumulates to high levels in tumor cells [79,80]. In addition, TP53 is prone to genetic mutations in cancer cells, which can disrupt its tumor-suppressive functions. Thus, it plays a regulatory role in defective cells and in the transformation of normal cells into cancer cells [39,81]. AKT is a core factor in the PI3K/AKT signaling pathway, comprising three members in the AKT family: AKT1/PKBα, AKT2/PKBβ, and AKT3/PKBγ. AKT1 regulates cell metabolism, apoptosis, proliferation, and glucose metabolism. Research has shown that AKT1 is closely linked to the differentiation, invasion, and metastasis of early-stage tumor cells. Therefore, inhibiting AKT1 activity may reduce tumor cell proliferation, promote tumor cell apoptosis, and disrupt the energy metabolism of cancer cells, thereby exerting anti-cancer effects [82,83]. According to the results of microarray data analysis, differential expression of the top 20 core genes was found across three different datasets retrieved from three GEO databases (GSE1111, GSE1111, and GSE11111). Survival analysis indicated that EGFR, TP53, and TNF were significantly associated with the survival and prognosis of liver cancer. These targets may represent specific receptors that CLYF interacts with in the human body during cancer treatment. Furthermore, CLYF may influence the secretion of specific substances by acting on these receptors to achieve its therapeutic effects.

GO and KEGG enrichment analyses revealed significant enrichment in numerous cancer-related GO functions and KEGG signaling pathways. A total of 128 pathways were identified in the KEGG enrichment analysis, including the PI3K–Akt signaling pathway, MAPK signaling pathway, hepatitis B signaling pathway, hepatitis C signaling pathway, and AGE–RAGE signaling pathway in diabetic complications. The PI3K–Akt signaling pathway modulates the cell cycle and can be targeted for cancer treatment [36]. Additionally, the PI3K–Akt pathway plays a crucial role in tumor occurrence, proliferation, cell cycle progression, and apoptosis, as well as in the epithelial–mesenchymal transition of tumors, contributing to drug resistance [81,83]. Importantly, both the PI3K–Akt and MAPK signaling pathways have also been reported to be closely related to liver cancer treatment [33,34,35,37,84,85]. The PI3K–Akt signaling pathway, MAPK signaling pathway, and AGE–RAGE signaling pathway in diabetic complications are primarily linked to oxidative stress, immune regulation, and the inflammatory response. Among these, the AGE–RAGE signaling pathway is particularly associated with inflammation, activating the MAPK pathway and interfering with immune and oxidative stress responses [37]. These signaling pathways ranked among the top 20 in the PPI network, providing a potential mechanism for treating liver cancer with CLYF. Therefore, the effective treatment of liver cancer using CLYF is predicated based on these core targets and pathways. Specifically, the therapeutic effects of CLYF on liver cancer are primarily achieved by interacting with specific receptors or enzymes to influence the secretion of core compounds.

This study constructed a compound–herb–key target interaction network (Figure 4) and a compound–target–pathway interaction network (Figure 6), which facilitated the identification of the most promising active compounds in CLYF and their putative therapeutic targets. The binding between core compounds and receptors was evaluated using molecular docking to assess binding energy. Specifically, this study narrowed our list to 32 core active components and 3 core therapeutic targets, predicting the binding of each compound to these 3 therapeutic targets through molecular docking. The results showed that these 32 active ingredients exhibited strong binding affinity toward EGFR (Table S5). With the exception of TM12 (sucrose) in TM, the remaining 31 core active compounds also demonstrated strong binding activity toward TP53 (Table S6) and AKT1 (Table S7). These molecular docking results align with the findings from our network pharmacological analysis.

To evaluate the effects of the CLYF formulation and the top six active compounds identified through binding energy predictions, in vitro cell experiments were performed. CLYF-A inhibited the cell proliferation of HepG2 cells, as did each of the six individual compounds. The percentage content of these six compounds in CLYF-A was determined via HPLC, aligning with the predictions made through network pharmacology and molecular docking. In addition, CLYF-A also induced cell apoptosis and cell cycle arrest in HepG2 cells, which was associated with the loss of mitochondrial membrane potential. These findings suggest that CLYF regulates the intersected targets and pathways through these core compounds, thereby exerting therapeutic effects on liver cancer.

The targets EGFR [77] and AKT1 [82,83] are proteins that promote cancer occurrence. Western blot experiments revealed that the relative protein expression levels of EGFR and AKT1 were reduced in HepG2 cells treated with the CLYF formula compared to the control group (Figure 14A,B,D). This suggests that the Naxi traditional formula CLYF may be effective for cancer treatment. Conversely, TP53 is a tumor-suppressor protein with a strong correlation with tumor activity [79,80]. In comparison with the control group, the relative protein expression levels of TP53 were increased after treatment with the CLYF formula in HepG2 cells (Figure 14A,C), indicating that the Naxi traditional formula CLYF may possess anti-cancer activity. In summary, the Western blot experiments further validated the prediction made through network pharmacology and molecular docking, providing molecular evidence that CLYF had potential anti-cancer effects.

The traditional Naxi CLYF formula with clinical application in folk medicine is reported for the first time regarding its composition and ratios, marking the primary objective in this study. The anti-cancer activity underlying the pro-apoptotic and cytotoxic effects of CLYF was verified using various scientific methods, including network pharmacology, microarray data analysis, survival analysis, molecular docking, in vitro experiments, and Western blot experiments. These approaches provide a research foundation and methodology for TCM formulas that have been clinically applied in folk practices. In this study, network pharmacology was employed to preliminarily predict the effective ingredients and potential targets of CLYF for treating liver cancer. Additionally, a multi-component and multi-target network was constructed to elucidate the complex interactions between active compounds and target proteins. Microarray analysis, survival analysis, and molecular docking served as molecular verification tools to further validate the predictive results regarding targets and pathways identified through network pharmacology. In vitro experiments—including cell proliferation assays, cell cycle and apoptosis assays, and mitochondrial membrane potential determination—were conducted to verify the anti-cancer activity underlying the pro-apoptotic and cytotoxic effects of CLYF. Western blot experiments were performed to assess the relative protein expression levels following treatment with CLYF-A, compared to the control group. However, the deeper molecular mechanism underlying CLYF remains unclear, necessitating further research through additional pharmacological experiments and in vivo experiments. Additionally, the current methods used in this study have some limitations, as the active compounds in the samples or formulas were only searched from databases or the literature, which restricts the range of discovered compounds to those that are already known. However, there may be other novel anti-liver cancer compounds in herbs or formulas that have yet to be discovered. Using metabolomics to analyze the main active compounds in herbs or formulas, combined with transcriptomics, proteomics, pharmacology, pharmacodynamics, pharmacokinetics, and other methods, to screen for more effective components and analyze of their molecular mechanisms, will be more convincing.

## 4. Materials and Methods

### 4.1. Medicinal Materials, Chemicals, Reagents, Cell Culture, and Treatment

The herbs that comprise CLYF were provided by Lijiang Yunxin Green Biological Development Co., Ltd. (Lijiang, China), in Northwestern Yunnan Province, China, in May 2022. The 11 herbs were stored in the Key Laboratory of Economic Plants and Biotechnology, Kunming Institute of Botany, Chinese Academy of Sciences, Kunming, China and identified in May 2022 according to their morphological features by Professor Heng Li (CAS Key Laboratory for Plant Diversity and Biogeography of East Asia, Kunming Institute of Botany, Chinese Academy of Sciences, China). The voucher number for each herb is shown in Table S1. Ethanol was analytical grade. A centrifuge (L530, Changsha High and New Technology Industrial Development Zone, Xiangyi Centrifuge Instrument Co., Ltd., Changsha, China) was used to separate solids from liquids in suspension. Quercetin (SQ7) was purchased from ShangHai D&B Biological Science and Technology Co. Ltd. (purity ≥ 97%, Shanghai, China). Solanidine (CBM16) was purchased from Beijing Wokai Biotechnology Co., Ltd. (purity ≥ 99%, Beijing, China). Diosgenin (CL2), pennogenin (CL3), ginsenoside Rh_2_ (CF3), and verticinone (CBM9) were purchased from Sichuan Weikeqi Biological Technology Co., Ltd. (purity ≥ 98%, Chengdu, China).

Human liver cancer cell lines (HepG2) were purchased from BNCC (BeNa culture Collection, Beijing, China) and cultured in DMEM medium (Procell Life Science & Technology Co., Ltd., Wuhan, China) supplemented with 10% (*v*/*v*) fetal bovine serum (Procell Life Science & Technology Co., Ltd.) and 1% (*v*/*v*) penicillin–streptomycin solution (stocks of 10,000 U/mL penicillin G sodium salt; 10 mg/mL streptomycin, Procell Life Science & Technology Co., Ltd.) in an incubator set to 37 °C and 5% (*v*/*v*) CO_2_.

### 4.2. CLYF Preparation and Extraction

The CLYF formula represented the clinical experience of the Naxi people in treating liver cancer, demonstrating significant therapeutic effects. This knowledge had been documented in local medical books written in the Naxi language and has been passed down through at least three generations. In order to prepare the powder that is the basis of the CLYF formulation, the dried parts of 11 herbs were crushed into powder with a pulverizer. The relative proportion of each herb is listed in Table S1. The extraction was conducted using traditional methods of CLYF consumption among the Naxi people, utilizing ethanol and deionized water as solvents [12]. The CLYF powder (50.0 g) was first extracted three times in 75% (*v*/*v*) ethanol (400 mL each extraction) and refluxed at 100 °C for 4 h. The filtrate was filtered through filter paper, merged, and concentrated by rotary evaporator as ethanol extract (CLYF-A, 12.1 g). The residual ethanol was allowed to evaporate naturally from the filtered residue, which was then extracted with deionized water (H_2_O, 3 × 400 mL) by refluxing at 100 °C for 4 h. The liquid portion was collected via centrifugation (4000 r/min, 10 min, 25 °C), combined, and concentrated using a rotary evaporator as the aqueous CLYF extract (CLYF-W, 8.9 g). The drugs were filtered, dried, and collected as the residual portion of CLYF (CLYF-S, 26.0 g) for further research.

### 4.3. Collection of Active Chemical Compounds and Their Corresponding Targets in CLYF

CLYF is composed of 11 herbs. The active chemical compounds in each herb were obtained from the TCMSP database (https://old.tcmsp-e.com/tcmsp.php (accessed on 4 May 2023)), BATMAN-TCM database (http://bionet.ncpsb.org.cn/batman-tcm/index.php/ (accessed on 4 May 2023)), and literature searches from Web of Science (https://www.webofscience.com/wos/alldb/basic-search (accessed on 4 May 2023)). In TCMSP, the ADME property value was set to Oral Bioavailability (OB) ≥ 30% and Drug Likeness (DL) ≥ 0.18. In BATMAN-TCM, the input parameter score cutoff was set to 20, and the adjusted *p*-value was 0.05. The chemical structures and SMILES (Simplified Molecular Input Line Entry System) code of these active compounds were downloaded from the PubChem database (https://pubchem.ncbi.nlm.nih.gov/ (accessed on 6 May 2023)). The Swiss Institute of Bioinformatics (SIB) database was used to perform ADME screening (http://www.swissadme.ch/ (accessed on 7 May 2023)). The ADME characteristics were Lipinski (yes), glucose absorption (high), the rotatable bonds (less than or equal to 5), the hydrogen-bond acceptors (less than 10), and the hydrogen-bond donors (less than 5). The compound targets were predicted with the Swiss Target Prediction tool (http://www.swisstargetprediction.ch/ (accessed on 7 May 2023)). The official gene names of all target genes were confirmed or corrected by querying the UniProt database (https://www.uniprot.org/ (accessed on 8 May 2023)).

### 4.4. Collection of Disease-Related Proteins and Therapeutic Targets

Therapeutic targets for treating liver cancer were collected from four databases: GeneCards (https://www.genecards.org/ (accessed on 12 May 2023)), Online Mendelian Inheritance in Man (OMIM, https://omim.org/ (accessed on 12 May 2023)), Therapeutic Target (TTD, http://db.idrblab.net/ttd/ (accessed on 12 May 2023)), and DrugBank (https://go.drugbank.com/ (accessed on 12 May 2023)). The repetitive region of gene targets was removed. The overlap of therapeutic targets across the four databases was illustrated using a Venn diagram. The official names of all therapeutic targets were confirmed or corrected by querying the UniProt database. Then, the overlap between the putative targets for each active ingredient and the therapeutic targets was determined using the online tool Venny (https://bioinfogp.cnb.csic.es/tools/venny/ (accessed on 19 May 2023)). The intersection between the two sets of proteins were considered potential targets for the CLYF formula in anti-cancer treatment.

### 4.5. Network Construction and Analysis

The putative CLYF targets for cancer treatment, as defined above, were entered into the String database (https://string-db.org/ (accessed on 24 May 2023)) to obtain a list of interacting proteins and assess the strength of the proposed interactions. The resulting file was then loaded into Cytoscape 3.7.1 software to define the underlying PPI network. The Network Analyzer tool within Cytoscape 3.7.1 was used to analyze the node degree, and the top 20 therapeutic targets and their associated core active ingredients of CLYF with anti-cancer properties were extracted. Cytoscape 3.7.1 software was used to construct the medicine–component–target diagrams.

### 4.6. GO and KEGG Pathway Enrichment Analyses

GO and KEGG pathway enrichment analyses were carried out on core therapeutic targets using the database Metascape (https://metascape.org (accessed on 14 June 2023)), with the species set to “*Homo sapiens*” and the *p*-value cutoff set to 0.01 [86]. The GO categories BP, CC, and MF and KEGG pathways were selected for enrichment analysis. The top 20 GO terms obtained from the BP, CC, and MF categories, as well as KEGG pathways, were selected based on their *p*-values. The results were visualized on the website https://www.bioinformatics.com.cn (accessed on 16 June 2023).

### 4.7. Microarray Data Analysis

Gene Expression Omnibus (GEO) database (https://www.ncbi.nlm.nih.gov/geo/, (accessed on 3 August 2024)) is a high-throughput gene expression database that includes microarray chips, second-generation sequencing data, and other forms of high-throughput genomic data [27]. Three microarray datasets, GSE136247, GSE76427, and GSE87630, were downloaded from the GEO database (accessed on 2 August 2024). Differentially expressed genes were screened using the “Limma” package of R language (R version 4.4.0). Differential expression information was obtained by adding annotations and deduplicating through the “dplyr” package of R language. The “ggplot2” package of R was used to construct volcano maps and visualize changes in gene expression. *p* < 0.05 was considered statistically significant. The microarray data analysis of the top 20 targets was performed to screen out genes that were significantly upregulated or downregulated in liver cancer compared with the normal group for later survival analysis.

### 4.8. Survival Analysis

The online tool for the Kaplan–Meier Plotter (https://kmplot.com/analysis/ (accessed on 3 August 2024)) was used for the survival analysis of hub genes and evaluation of the clinical importance of a particular gene based on gene expression levels. The type of disease was selected as liver cancer and the organism was set to “*Homo sapiens*”. Kaplan–Meier survival curves were constructed for survival analysis and examining the relationship between core genes and overall survival of liver cancer patients. *p* < 0.05 was considered statistically significant. The impact of high or low expression of the core genes on liver cancer patients was analyzed by examining changes in gene expression levels in relation to clinical survival.

### 4.9. Molecular Docking Between CLYF Active Compounds and Their Target Proteins

Molecular docking was conducted to assess the interaction between target proteins and the active compounds in CLYF [22]. The 3D structure data (SDF files) of key active components in CLYF were downloaded from the PubChem database (https://pubchem.ncbi.nlm.nih.gov/ (accessed on 28 June 2023)). Subsequently, all SDF files were transformed to a MOL2 file format using Open Babel 3.1.1 software (https://openbabel.org/ (accessed on 28 June 2023)). Finally, the active compounds were pre-processed with full hydrogenation, designated as ligands, and automatically assigned a charge. The detection and configuration of rotatable bonds were completed, and the files were saved in PDBQT format for storage and further use.

The protein structures of the epidermal growth factor receptor (EGFR) [87], cellular tumor antigen p53 (TP53) [88], and RAC-alpha serine/threonine-protein kinase (AKT1) [89] were downloaded from the Research Collaboratory for Structural Bioinformatics (RCSB) Protein Data Bank (PDB) (https://www.rcsb.org/ (accessed on 6 July 2023)). The organism was specified as “*Homo sapiens*” and the resolution was set to less than 2.5 Å. Original water molecules and small molecular ligands were removed using PyMol 2.4.0 software, and the files were saved in PDB format. To complete the receptor selection, hydrogens and total Gasteiger charges were added, and the files were saved in PDBQT format for molecular docking analysis. The Autogrid was employed to set the grid box to encompass the entire molecule to identify the docking active center. After defining the binding sites of the targets, the first docking calculation was set using the Autodock 4.2.6 software. The method of Local Search Parameters was used to calculate the second docking through Autodock. Finally, all binding energies were quantified to verify the predicted results of network pharmacology, with lower binding energies indicating better binding activity between the target proteins and core active compounds. The final files were saved in PDBQT format for further visualization using PyMol 2.4.0 software.

### 4.10. Determination of Contents of Six Core Active Compounds 

Samples were dissolved in methanol and filtered through a 0.45 μm membrane before use for HPLC analysis. Six core active compounds (SQ7, CL2, CL3, CF3, CBM9, CBM16) were divided into two groups to analyze and determine the content in the extract for CLYF-A according to the compound property via HPLC (Agilent 1260 series system, Zorbax SB-C18 column, 5 μm, ϕ 4.6 × 250 mm). Four compounds (SQ7, CL2, CL3, and CF3) were analyzed via HPLC-DAD (High Performance Liquid Chromatography-Diode Array Detection) and prepared at concentrations of 0.2695, 0.2719, 0.2681, and 0.2703 mg/mL. Two compounds (CBM9 and CBM16) were analyzed via HPLC-ELSD (High Performance Liquid Chromatography-Evaporative Light Scattering Detector) and prepared at concentrations of 0.5401 and 0.5389 mg/mL.

The mobile phase for SQ7 consisted of (B1) methanol and (A1) 0.3% formic acid in water (*v*/*v*), with a flow rate of 1 mL/min, at 30 °C, with an injection volume of 10 μL, with the following gradient: 0–10 min, 10–20% B1; 10–25 min, 20–25% B1; 25–30 min, 25% B1; 30–35 min, 25–50% B1; 35–40 min, 50% B1; 40–45 min, 50–80% B1; 45–48 min, 80–0% B1. The mobile phase for three compounds (CL2, CL3, and CF3) consisted of (B2) acetonitrile and (A2) water, with a flow rate of 1 mL/min, at 30 °C, with an injection volume of 10 μL, with the following gradient: 0–20 min, 20% B2; 20–40 min, 20–95% B2; 40–65 min, 95% B2. The mobile phase for CBM9 consisted of (B2) acetonitrile and (A3) 0.03% triethylamine in water (*v*/*v*), with a flow rate of 1 mL/min, at 30 °C, with an injection volume of 2 μL, with the following gradient: 0–40 min, 70% B2. Both the evaporator and nebulizer temperature of ELSD were 40 °C, and the gas flow rate was 1.4 L/min. The mobile phase for CBM16 consisted of (B2) acetonitrile and (A4) 0.1% trifluoroacetic acid in water (*v*/*v*), with a flow rate of 1 mL/min, at 35 °C, with an injection volume of 10 μL, with the following gradient: 0–10 min, 5–100% B2; 10–13 min, 100% B2. Both the evaporator and nebulizer temperature of ELSD were 75 °C, and the gas flow rate was 2.5 L/min.

### 4.11. Cell Proliferation Assay

The effects of the CLYF extracts on cell proliferation were evaluated using the CCK-8 (Cell Counting Kit-8) assay on human liver cancer cell lines [90,91,92,93,94]. Each well of a 96-well plate (Corning Life Sciences [Wujiang] Co., Ltd., Suzhou, China) was seeded with 90 μL of cell culture medium (adherent cells 5 × 10^4^/mL and suspension cells 9 × 10^4^/mL) per well and then cultured at 37 °C and 5% (*v*/*v*) CO_2_ for 24 h. The cell suspension contained 10% (*v*/*v*) fetal bovine serum (Procell Life Science & Technology Co., Ltd.). CLYF extracts (10 μL) were added to each well at the following concentrations, each in three replicates: CLYF-A (0.1, 0.5, 1.0, 5.0, 10.0, 20 mg/mL) and DOX (0.001, 0.01, 0.1, 1.0, 5.0, 10.0 μM, Solarbio, Beijing, China) were dissolved in dimethyl sulfoxide (DMSO, Sigma, Shanghai, China); CLYF-W (0.1, 0.2, 0.5, 1.0, 2.0, 5.0 mg/mL) was dissolved in sterile water. The following six compounds were dissolved in DMSO: CBM9 (1.0, 10.0, 50.0, 100.0, 200.0, 500.0 μM), CBM16 (0.01, 0.1, 10.0, 50.0, 200.0, 500.0 μM), SQ7 (0.1, 1.0, 50.0, 100.0, 200.0, 500.0 μM), CL2 and CL3 (0.1, 10.0, 50.0, 100.0, 200.0, 500.0 μM), CF3 (0.01, 1.0, 50.0, 100.0, 200.0, 500.0 μM). Here, a 1.0 μM compound is equivalent to 1.0 × M × 10^−6^ mg/mL. M is the molecular weight of the compound in g/mol. After culturing for 48 h in an incubator at 37 °C and 5% (*v*/*v*) CO_2_, the old culture medium and drug solution were removed from adherent cells, and 100 μL of CCK-8 solution (diluted 10 times with alkaline medium) was added. For suspension cells, 10 μL of CCK-8 stock solution was directly added to each well. After an incubation of 1–4 h in the dark and real-time observations, the absorbance at 450 nm was measured using a microplate reader (Multiskan MK3, Thermo, Shanghai, China). The IC_50_ value was calculated with Graphpad Prism 8 (version 8.0.2, from GraphPad Software Inc., La Jolla, CA, USA). DOX was used as a positive control in the cell experiments.

### 4.12. Cell Cycle and Apoptosis Assays

Flow cytometry (BeckmanCytoFLEX, Beckman, Pasadena, LA, USA) was used to measure the effects of CLYF on cell cycle and apoptosis by staining with PI (propidium iodide, Beyotime Biotech. Inc., Shanghai, China) and annexin V-FITC/PI (annexin V-fluorescein isothiocyanate/propidium iodide, Beyotime Biotech. Inc., Shanghai, China), respectively. HepG2 cells were cultivated in six-well plates at a density of 10 × 10^5^ cells per well for 24 h before the treatment with different concentrations of CLYF-A (0.125, 0.25, 1.0 mg/mL) and the positive control (0.5 μM) for 24 h. Doxorubicin (DOX, Solarbio) was used as the positive control, which was the chemotherapy drug in cancer treatment. Adherent cells were treated with trypsin digestion fluid (Cell Cycle and Apoptosis Analysis Kit, Beyotime Biotech. Inc., Shanghai, China). After that, all cells were collected, centrifuged, and washed with phosphate-buffered saline (PBS). For the determination of cell cycle, cells were fixed in 70% (*v*/*v*) ice-cold ethanol at 4 °C for 2 h and then centrifuged for 5 min to precipitate cells and washed again in pre-ice-cold PBS. The cells were stained with 0.5 mL of PI for each tube at 37 °C for 30 min. The cell cycle stage was analyzed via flow cytometry. Red fluorescence at a 488 nm excitation wavelength and light scattering were detected. For the determination of cell apoptosis, the cells were incubated with 195 μL of binding buffer of annexin V-FITC, after which 5 μL of annexin V–FITC and 10 μL PI were added at 37 °C for 20 min before determination via flow cytometry.

### 4.13. Determination of Mitochondrial Membrane Potential

Twenty-four-well white transparent bottom cell plates were seeded with 1 × 10^5^ cells per well and cultured for 24 h. The cultured medium was removed. And then the cells were treated with different concentrations of CLYF-A (0.125, 0.25, 0.5 mg/mL) and the positive control (DOX, 0.5 μM) for 24 h. After removing the cultured medium, the cells were added to 0.5 mL of JC-1 dyeing solution (Annexin V-FITC Apoptosis Detection Kit, Beyotime Biotech. Inc., Shanghai, China) at 37 °C for 20 min. The supernatant was removed after incubation, and the cells were washed twice with 1 mL of JC-1 buffer (×100). Cell culture medium (0.5 mL) was added to each well, and the stained cells were observed with an inverted fluorescence microscope (IX70, Olympus, Tokyo, Japan). The wavelengths for excitation were 490 nm (monomers for JC-1) and 525 nm (polymer for JC-1). And the wavelengths for emission were 530 nm (monomers for JC-1) and 590 nm (polymer for JC-1).

### 4.14. Western Blot Assay

HepG2 cells were cultured in six-well plates at a density of 5 × 10^5^ cells per well at 37 °C and 5% (*v*/*v*) CO_2_ for 24 h. The ethanol extract of CLYF-A (0.25 mg/mL) for the traditional Naxi formula was used to intervene in HepG2 cells in three replicates for another 24 h. The same concentration of DMSO was used as a negative control, referred to as the control group. β-Actin served as the internal reference gene. After removing the cultured medium and washing with pre-ice-cold PBS, 100 μL of prepared RIPA lysate (added with 1 μL protease inhibitor and 1 μL phosphatase inhibitor, Servicebio, -Wuhan, China) was added to each well. The prepared RIPA lysate needed to be shaken and placed on ice and used within 0.5 h. After the system was placed on ice and allowed to lyse for 10 min, the supernatant was obtained via centrifugation, which contained the total protein.

The BCA kit (GBCBIO Technologies Inc., Guangzhou, China) was used to determine the protein concentration in the collected sample. Here, 5× reduced protein loading buffer (Servicebio, Wuhan, China) was added to the extracted protein solution at a ratio of 4:1 (*v*/*v*) and denatured in boiling water bath for 10 min. Then, 40 μg of protein was added to each well in an electrophoresis tank. The protein was separated via SDS-PAGE gel electrophoresis and then transferred to a PVDF membrane. After sealing with 5% skimmed milk powder for 2 h, the primary antibodies for EGFR (1:1000), TP53 (1:1000), AKT1 (1:2000), and β-Actin (1:20,000) were added according to the antibody instructions to soak and incubate the PVDF membrane overnight at 4 °C. The PVDF membrane was washed five times with TBST (Servicebio, Wuhan, China) for five min each time. The secondary antibody was then added to incubate for 1 h at room temperature. TBST was added again, and the PVDF membrane was washed five times for five min each time. The ECL reagent (Servicebio, Wuhan, China) and development fixer reagent kit (Tianjin Hanzhong Photography Materials Factory, Tianjin, China) were used for color exposure according to the instructions. After the photographic film was scanned and dried, IPP 6.0.0.260 software was used to analyze the grayscale values.

### 4.15. Statistical Analysis

All data are presented as means ± standard deviations (SDs) from three parallel experiments. A *t*-test was used to evaluate the statistical analysis in comparing two groups for the data of Western blotting using Excel 2019. One-way ANOVA was used to evaluate significant differences in multiple groups followed by LSD, Tukey’ s-b, and Waller–Duncan tests using SPSS software (Version 26, IBM, Armonk, NY, USA). *p* < 0.05 was considered statistically significant.

## 5. Conclusions

This study validated the key components, target therapeutic proteins, and signaling pathways through network pharmacology, microarray data analysis, survival analysis, molecular docking, in vitro experiments, and Western blot experiments. Firstly, the network pharmacology approach was used to predict the effective ingredients and potential targets of CLYF against liver cancer, primarily focusing on EGFR, TP53, and AKT1. This method allowed for the construction of a multi-component and multi-target network, which better elucidated the complex interactions between the active compounds and target proteins. Secondly, CLYF tends to treat liver cancer through the PI3K–Akt signaling pathway, MAPK signaling pathway, hepatitis B signaling pathway, and hepatitis C signaling pathway to exert drug effects. With the help of microarray data analysis, survival analysis, and molecular docking, we conducted further molecular verification of the predictive targets and pathways identified through network pharmacology. In addition, cell experiments indicated that CLYF-A suppressed cell proliferation and induced cell apoptosis and cell cycle arrest in HepG2 cells, which were associated with the loss of mitochondrial membrane potential. Finally, the results of Western blotting showed that the potential anti-cancer mechanism of the traditional Naxi formula CLYF may be related to inhibiting the relative protein expression of EGFR and AKT1 while promoting the relative protein expression of TP53. In summary, the anti-cancer activity underlying the pro-apoptotic and cytotoxic effects of the Naxi formula CLYF was systematically verified through scientific methods, including network pharmacology, microarray data analysis, survival analysis, molecular docking, in vitro experiments, and Western blot experiments, which is based on Naxi indigenous knowledge. This study offered new possibilities for clinical treatments and CLYF formulations. Additionally, it provided methodologies and case support for the scientific validation of the efficacy of various ethnic folk formulas. Of course, there are some limitations to this study: firstly, the deeper molecular mechanisms underlying the action of CLYF need more pharmacological experiments. Secondly, this study did not test the efficacy or safety of CLYF in vivo. Finally, more anti-tumor cases, toxic effects, and adverse events should be analyzed in clinical studies.

## Figures and Tables

**Table 1:** List of the top 20 pathways showing enrichment based on KEGG analysis.

Rank	Pathways	Number of Genes	*p*-Value
1	hsa05200: Pathways in cancer	17	1.21 × 10^−27^
2	hsa05205: Proteoglycans in cancer	14	1.03 × 10^−26^
3	hsa05163: Human cytomegalovirus infection	14	3.94 × 10^−26^
4	hsa05167: Kaposi sarcoma-associated herpesvirus infection	13	1.54 × 10^−24^
5	hsa05207: Chemical carcinogenesis–receptor activation	12	1.23 × 10^−21^
6	hsa05210: Colorectal cancer	10	3.63 × 10^−21^
7	hsa05224: Breast cancer	11	3.95 × 10^−21^
8	hsa05160: Hepatitis C	11	8.33 × 10^−21^
9	hsa04933: AGE-RAGE signaling pathway in diabetic complications	10	1.77 × 10^−20^
10	hsa05213: Endometrial cancer	9	3.02 × 10^−20^
11	hsa05417: Lipid and atherosclerosis	11	2.87 × 10^−19^
12	hsa01521: EGFR tyrosine kinase inhibitor resistance	9	5.80 × 10^−19^
13	hsa05219: Bladder cancer	8	6.86 × 10^−19^
14	hsa05161: Hepatitis B	10	2.59 × 10^−18^
15	hsa05215: Prostate cancer	9	4.00 × 10^−18^
16	hsa04010: MAPK signaling pathway	11	9.44 × 10^−18^
17	hsa04510: Focal adhesion	10	2.59 × 10^−17^
18	hsa04919: Thyroid hormone signaling pathway	9	3.14 × 10^−17^
19	hsa05165: Human papillomavirus infection	11	3.52 × 10^−17^
20	hsa04151: PI3K-Akt signaling pathway	11	7.40 × 10^−17^

**Table 2:** Compounds showing the lowest molecular docking energies with their predicted targets.

NO.	Target	PDB ID	Protein Name	Compound Name	Binging Energy (kcal/mol)
1	EGFR	1XKK	CBM9	Verticinone	−6.36
2			CBM16	Solanidine	−7.65
3			ZJ2	Rosenonolactone	−7.07
4	TP53	8DC6	CBM9	Verticinone	−4.01
5			CBM16	Solanidine	−5.03
6			ZJ2	Rosenonolactone	−3.60
7	AKT1	6NPZ	CBM9	Verticinone	−3.92
8			CBM16	Solanidine	−4.28
9			ZJ2	Rosenonolactone	−3.97

**Table 3:** IC_50_ values for CLYF extracts and six core active compounds against HepG2 cells.

No.	Extract or Compound	Compound Name	IC_50_ ± SD
CLYF-A	Crude extract (mg/mL)	Not applicable	0.25 ± 0.03e
CLYF-W			>5
CBM9	Compound (μM)	Verticinone	76.44 ± 0.88d
CBM16		Solanidine	128.17 ± 16.00d
SQ7		Quercetin	194.97 ± 5.31b
CL2		Diosgenin	244.80 ± 0.79a
CL3		Pennogenin	151.23 ± 9.32c
CF3		Ginsenoside Rh_2_	74.78 ± 2.48d
DOX	Positive control (μM)	Doxorubicin	0.15 ± 0.03e

Note: Different lowercase letters (a, b, c, d, and e) indicate significant differences (*p* < 0.05).

**Table 4:** The percentages of HepG2 cells in G0/G1, S, and G2/M phases displayed based on flow cytometry.

	G0/G1 (%)	S (%)	G2/M (%)
Control	68.24 ± 0.93a	22.15 ± 0.84a	9.62 ± 0.17d
0.125 mg/mL	66.77 ± 0.65a	22.21 ± 0.15a	11.02 ± 0.51d
0.25 mg/mL	62.76 ± 1.36b	20.62 ± 0.93b	16.62 ± 1.43c
1.0 mg/mL	60.76 ± 1.42b	19.16 ± 0.39c	20.08 ± 1.09b
DOX	5.34 ± 0.57c	1.80 ± 0.26d	92.87 ± 0.37a

Note: data are presented as means ± SDs from three repeated experiments. These data were statistically processed, and different letters (a, b, c, and d) indicate significant differences at different concentrations (*p* < 0.05).

**Table 5:** The percentages of HepG2 cells in each quadrant fractions displayed based on flow cytometry.

	Live Cells (%)	Early Apoptosis (%)	Late Apoptosis (%)
Control	84.60 ± 0.92a	11.97 ± 0.71e	2.89 ± 0.31c
0.125 mg/mL	81.83 ± 0.32b	14.93 ± 0.38d	2.78 ± 0.08c
0.25 mg/mL	71.93 ± 0.64c	24.43 ± 0.23b	3.50 ± 0.37b
1.0 mg/mL	33.03 ± 1.21d	64.03 ± 1.19a	2.83 ± 0.16c
DOX	70.83 ± 0.64c	21.67 ± 1.25c	6.02 ± 0.31a

Note: data are presented as means ± SDs from three repeated experiments. These data were statistically processed, and different letters (a, b, c, d, and e) indicate significant differences at different concentrations (*p* < 0.05).

## Data Availability

The original contributions presented in the study are included in the article/Supplementary Materials. Supporting information and further inquiries can be directed to the corresponding author.

## Supplementary Materials

The following supporting information can be downloaded at: https://www.mdpi.com/article/10.3390/ph17111429/s1, Table S1: Composition of CLYF. Table S2: Active ingredients in CLYF. Table S3: Functional information of the top 20 targets. Table S4: Top 35 active ingredients in CLYF. Table S5: Molecular docking energy of EGFR (PDB ID 1XKK). Table S6: Molecular docking energy of TP53 (PDB ID 8DC6). Table S7: Molecular docking energy of AKT1 (PDB ID 6NPZ). Table S8: Statistical analysis of relative expression level of the core targets EGFR, TP53, and AKT1 in HepG2 cells after treatment with CLYF-A. Figure S1: Network of herb–compound–targets. Herbs are shown in red; putative therapeutic targets of CLYF are shown in green; CF1 to CF11 are 11 redundant compounds in 11 herbs and are shown in purple; other colors indicate all compounds found in the 11 herbs that constitute the CLYF formulation. Edges (lines between nodes) represent the interactions between herbs and compounds or between compounds and targets. Figure S2: Interaction network of the top 20 targets of CLYF. The green nodes represent the top 20 hub genes; the blue nodes represent the pathways related to the hub targets. Figure S3: Three-dimensional visualization of molecular docking between the core therapeutic targets and the corresponding active compound of CLYF. (A) EGFR and CBM9. (B) EGFR and CBM16. (C) EGFR and ZJ2. (D) TP53 and CBM9. (E) TP53 and CBM16. (F) TP53 and ZJ2. (G) AKT1 and CBM9. (H) AKT1 and CBM16. (I) AKT1 and ZJ2. Figure S4: Inhibitory effect of CBM9 (A), CBM 16 (B), SQ7 (C), CL2 (D), CL3 (E), and CF3 (F) on HepG2 cells. Different lowercase letters indicate significant differences (*p* < 0.05). Figure S5: Figures for the original data of the Western blot experiments repeated three times independently in the article. (A) EGFR. (B) TP53. (C) AKT1. (D) β-Actin.

## Abbreviations

WHO, World Health Organization; HCC, Hepatocellular carcinoma; CLYF, Chong-Lou-Yao-Fang; TCM, traditional Chinese medicine; LCS, Le-Cao-Shi; PPI, Protein–protein interaction; TCMSP, Traditional Chinese Medicine Systems Pharmacology Database and Analysis Platform; BATMAN-TCM, Bioinformatics Analysis Tool for Molecular Mechanism of Traditional Chinese Medicine; ADME, Absorption, Distribution, Metabolism, and Excretion; OB, Oral Bioavailability; DL, Drug Likeness; GO, Gene Ontology; KEGG, Kyoto Encyclopedia of Genes and Genomes; BP, Biological Process; CC, Cellular Component; MF, Molecular Function; RCSB, Research Collaboratory for Structural Bioinformatics; PDB, Protein Data Bank; CL, *Paris polyphylla* var. *yunnanensis* (Franch.) Hand.-Mazz.; ZZS, *Panax bipinnatifidus* Seem.; SQ, *Panax notoginseng* (Burkill) F. H. Chen ex C. H. Chow; CBM, *Fritillaria cirrhosa* D. Don; DSL, *Pleione bulbocodioides* (Franch.) Rolfe; JTS, *Psammosilene tunicoides* W. C. Wu & C. Y. Wu; XYS, *Panax quinquefolius* L.; ZJ, *Engleromyces sinensis* M.A. Whalley, Khalil, T.Z. Wei, Y.J. Yao & Whalley; QYS, *Cynanchum otophyllum* C. K. Schneid.; GC, *Glycyrrhiza yunnanensis* S. H. Cheng & L. K. Dai ex P. C. Li; TM, *Gastrodia elata* Bl.; DOX, Doxorubicin. IC50, half maximal inhibitory concentration. SD, standard deviation. DMSO, Dimethyl sulfoxide; HPLC, High Performance Liquid Chromatography. HPLC-DAD, High Performance Liquid Chromatography-Diode Array Detection. HPLC-ELSD, High Performance Liquid Chromatography-Evaporative Light Scattering Detector. CCK-8, Cell Counting Kit-8.
