# Supplementary material for: An Investigation of the Effect of the Traditional Naxi Herbal Formula Against Liver Cancer Through Network Pharmacology, Molecular Docking, and In Vitro Experiments
Source: Pharmaceuticals (Basel). 2024 Oct 25;17(11):1429. doi: 10.3390/ph17111429 (PMC11597843; doi:10.3390/ph17111429)
Supplement: Supplementary file 1 [file pharmaceuticals-17-01429-s001.zip › pharmaceuticals-3266247-supplementary.pdf]

Table S1. Composition of CLYF.

No.	Abbreviation	Chinese name	Scientific name	Family	Tissue	Voucher number	Ratio (%)
1	CL	Dian chong lou	*Paris polyphylla* var. *yunnanensis* (Franch.) Hand.-Mazz.	Melanthiaceae	Rhizome	PF001	32.6
2	ZZS	Ge da qi	*Panax bipinnatifidus* Seem.	Araliaceae	Rhizome	PF002	16.3
3	SQ	San qi	*Panax notoginseng* (Burkill) F. H. Chen ex C. H. Chow	Araliaceae	Root and rhizome	PF003	5.4
4	CBM	Chuan bei mu	*Fritillaria cirrhosa* D. Don	Liliaceae	Bulb	PF004	10.9
5	DSL	Du suan lan	*Pleione bulbocodioides* (Franch.) Rolfe	Orchidaceae	Pseudo bulb	PF005	5.4
6	JTS	Jin tie suo	*Psammosilene tunicoides* W. C. Wu & C. Y. Wu	Caryophyllaceae	Root	PF006	1.1
7	XYS	Xi yang shen	*Panax quinquefolius* L.	Araliaceae	Root	PF007	5.4
8	ZJ	Zhu jun	*Engleromyces sinensis* M.A. Whalley, Khalil, T.Z. Wei, Y.J. Yao & Whalley	Xylariaceae	Fruit body	PF008	1.1
9	QYS	Qing yang shen	*Cynanchum otophyllum* C. K. Schneid.	Apocynaceae	Root	PF009	5.4
10	GC	Yun nan gan cao	*Glycyrrhiza yunnanensis* S. H. Cheng & L. K. Dai ex P. C. Li	Fabaceae	Root	PF010	5.4
11	TM	Tian ma	*Gastrodia elata* Bl.	Orchidaceae	Tuber	PF011	10.9

Table S2. Active ingredients in CLYF.

NO. ID	Scientific name of TCM	Chemical name of active ingredient
CL1	*Paris polyphylla* var. *yunnanensis* (Franch.) Hand.-Mazz.	4-aminobutyric acid
CL2		Diosgenin
CL3		Pennogenin
CL4		Trillin
CL5		20-hydroxyecdysone
ZZS1	*Panax bipinnatifidus* Seem	4'-hydroxywogonin
ZZS2		3,4,5-trimethoxybenzoic acid
ZZS3		2,6-dimethoxyphenol
SQ1	*Panax notoginseng* (Burkill) F. H. Chen ex C. H. Chow	Mandenol
CF1		Liquiritigenin
SQ3		Diisooctyl phthalate
CF2		beta-sitosterol
SQ5		Stigmasterol
CF3		Ginsenoside Rh2
SQ7		Quercetin
SQ8		Tetradecane
SQ9		Tridecene
SQ10		1,22-docosanediol
CF4		Ginsenoside
SQ12		(-)-2d,4d,6d,8d-tetramethyl Undecanoic Acid
SQ13		Sandaracopimarinol
SQ14		Dicapryl phthalate
SQ15		delta-elemene
SQ16		Cyperene
SQ17		(−)-trifara-9,14-Diene
SQ18		Cycloeucalenol
SQ19		4,4'-diketo-3-hydroxy-beta-carotene
SQ20		Nonadecanoic acid
SQ21		Ditertbutyl phthalate
CF5		Hexadecanoic acid
CF6		Dauricine
SQ24		3,4-dimethylbenzoic acid
SQ25		epsilon-cadinene
SQ26		1-tetradecanol
SQ27		Cyperol
SQ28		1-heptadecanol
CF7		N-nonanol
SQ30		Acetophenone
CF8		Encecalin
CF9		20-hexadecanoylingenol
SQ33		Elemicin
SQ34		Heneicosanic acid
SQ35		Coprine
SQ36		1,1,5,5-tetramethyl-4-Methano-2,3,4,6,7,10-Hexahydronaphthalene
SQ37		Delta-guaiene
SQ38		1-methyl-4-isoallyl-cyclohexane

CF10		Gamma-sitosterol
SQ40		Pentadecanoic acid
SQ41		Cuparene
CBM1	*Fritillaria cirrhosa* D. Don	bis[(2R)-2-ethylhexyl] benzene-1,2-dicarboxylate
CF2		beta-sitosterol
CBM3		Sitosterol
CBM4		Peimisine
CBM5		Cyclopamine
CBM6		isoverticine
CBM7		Korseveriline
CBM8		Korseverinine
CBM9		Verticinone
CBM10		Sinpemine A
CBM11		Menthyl acetate
CBM12		Methyl behenate
CBM13		Kosamol A
CBM14		Solanine
CF11		Methyl palmitate
CBM16		Solanidine
CBM17		Imperialine
CBM18		Methyl myristate
CBM19		Korsevinine
DSL1	*Pleione bulbocodioides* (Franch.) Rolfe	Colchicine
DSL2		Batatasin III
DSL3		Shanciol F
DSL4		Blestriarene A
DSL5		Pleionesin C
DSL6		Shanciol H
DSL7		Colchine
JTS1	*Psammosilene tunicoides* W. C. Wu & C. Y. Wu	Psammosilenin A
XYS1	*Panax quinquefolius* L.	(1S,2R,7R)-1-[(2R,3S)-3-heptyloxiran-2-yl]non-8-en-3,5-diyne-1,2,7-triol
XYS2		(8S,9S,10R,13R,14S,17R)-17-[(1R,4R)-4-ethyl-1,5-dimethylhexyl]-10,13-dimethyl-1,2,8,9,11,12,14,15,16,17-decahydrocyclopenta[a]phenanthren-7-one
CF9		20-hexadecanoylingenol
CF2		beta-sitosterol
CF3		Ginsenoside rh2
XYS6		Stigmast-7-enol
XYS7		Papaverine
XYS8		Sitogluside
XYS9		Daturilin
XYS10		4-phenylundecane
XYS11		Alpha-Farnesene
XYS12		6-phenyldodecane
XYS13		Octanol
XYS14		3-phenyldecane
CF7		N-nonanol
CF4		Ginsenoside

XYS17		3-phenylundecane
XYS18		Phlegmariuine-N
CF8		Encecalin
XYS20		5-phenyldodecane
XYS21		Caprylic acid
XYS22		6-phenylundecane
XYS23		5-phenyltridecane
XYS24		Pulegone
XYS25		4-phenyltridecane
XYS26		2-phenyldodecane
XYS27		3-phenyldodecane
XYS28		16-oxoseratenediol
XYS29		Beta-bisabolene
XYS30		Phenyl-2-propanone
XYS31		Calarene
XYS32		3-octanol
CF5		Hexadecanoic acid
XYS34		Alpha-guriunene
XYS35		5-phenyldolecane
XYS36		4-phenyldodecane
XYS37		1-phenylhexane
XYS38		Beta-curcumene
XYS39		Methyl caprylate
XYS40		Isofernene
ZJ1		Cytochalasin D
ZJ2		Rosenonolactone
ZJ3		19,20-epoxycytochalasin D
ZJ4		Trichothecin
ZJ5	*Engleromyces sinensis* M.A. Whalley, Khalil, T.Z. Wei, Y.J. Yao & Whalley	Demethylincisterol A2
ZJ6		Jiangxienone
ZJ7		Ergosterol peroxide
ZJ8		3,5,9-trihydroxyergosta-7,22-dien-6-one
ZJ9		1H-indole-3-carboxylic acid
ZJ10		Cytochalasin C
ZJ11		Epoxycytochalasin D
ZJ12		Cerevisterol
QYS1		4-hydroxyacetophenone
QYS2		2',4'-dihydroxy-6'-methoxyacetophenone
QYS3		Baishouwubenzophenone
CF11		Methyl palmitate
QYS5	*Cynanchum otophyllum* C. K. Schneid.	Vanillic acid
QYS6		Methyl 2,5-dihydroxybenzoate
QYS7		Vanillin
QYS8		Glycosmisic acid
QYS9		Arjunolic acid
GC1		18-beta-glycyrrhetinic acid
GC2	*Glycyrrhiza yunnanensis* S. H. Cheng & L. K. Dai ex P. C. Li	Glycyrrhetinic acid
GC3		Ruvoside
GC4		Corylifolinin

GC5		18-alpha-glycyrrhetinic acid
GC6		4'-O-methylglabridin
GC7		Isoliensinine
GC8		Dimethyl sebacate
GC9		Isotrilobine
GC10		Narwedine
GC11		Tetrahydroharmine
GC12		2-methyl-1,3,6-trihydroxyanthraquinone
GC13		Gancaonin I
GC14		Methylglyoxal
GC15		Hispaglabridin A
GC16		Glycyrrhetol
GC17		2,4,4'-trihydroxychalcone
GC18		3'-methoxyglabridin
GC19		Alpha-trihydroxy coprostanic acid
GC20		Licoricidin
GC21		Hispaglabridin B
GC22		Urea
GC23		Licobenzofuran
GC24		Phaseollinisoflavan
GC25		Methyl-24-hydroxyglycyrrhetate
GC26		Glyuranolide
GC27		5,6,7,8-tetrahydro-4-methylquinoline
GC28		Gancaonin E
GC29		Licocoumarone
GC30		Isoliquiritigenin
GC31		Ganoderic acid A
GC32		Sigmoidin B
GC33		Umbelliferone
CF10		gamma-sitosterol
GC35		Gmelofuran
CF1		Liquiritigenin
GC37		3-methyl-6,7,8-trihydropyrrolo[1,2-A] pyrimidin-2-one
GC38		Licoleafol
GC39		Tetrahydropalmatine
TM1		4-(4'-hydroxybenzyloxy) benzyl methyl ether
TM2		P-hydroxybenzaldehyde
CF10		gamma-sitosterol
TM4		P-hydroxybenzyl alcohol
TM5		M-hydroxybenzoic acid
CF9		20-hexadecanoylingenol
TM7		Gaultheroside A
CF6	*Gastrodia elata* Bl.	Dauricine
TM9		Bis(4-hydroxybenzyl) ether
TM10		4-ethoxymethylphenyl-4’-hydroxybenzylether
TM11		Vanillyl alcohol
TM12		Sucrose
TM13		Citronellal
TM14		4-hydroxybenzylamine

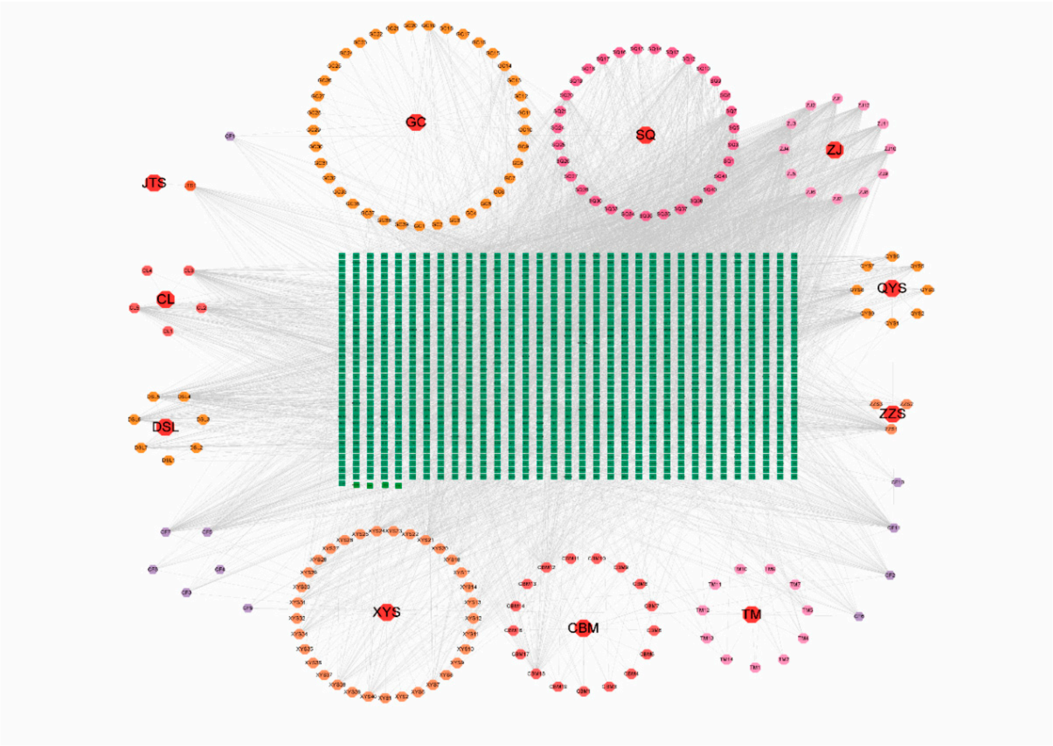

Figure S1. Network of herb–compound–targets. Herbs are shown in red; putative therapeutic targets of CLYF are shown in green; CF1 to CF11 are 11 redundant compounds in 11 herbs and are shown in purple; other colors indicate all compounds found in the 11 herbs that constitute the CLYF formulation. Edges (lines between nodes) represent the interactions between herbs and compounds or between compounds and targets.

Table S3. Functional information of the top 20 targets.

Rank	Target name	Description
1	AKT1	RAC-alpha serine/threonine-protein kinase
2	TP53	Cellular tumor antigen p53
3	TNF	Tumor necrosis factor
4	IL6	Interleukin-6
5	CTNNB1	Catenin beta-1
6	SRC	Proto-oncogene tyrosine-protein kinase Src
7	MYC	Myc proto-oncogene protein
8	EGFR	Epidermal growth factor receptor
9	VEGFA	Vascular endothelial growth factor A
10	JUN	Transcription factor Jun
11	MAPK3	Mitogen-activated protein kinase 3
12	IL1B	Interleukin-1 beta
13	STAT3	Signal transducer and activator of transcription 3
14	HSP90AA1	Heat shock protein HSP 90-alpha
15	CASP3	Caspase-3
16	PTEN	Phosphatidylinositol 3,4,5-trisphosphate 3-phosphatase and dual-specificity protein phosphatase PTEN
17	ESR1	Estrogen receptor
18	HIF1A	Hypoxia-inducible factor 1-alpha
19	EGF	Pro-epidermal growth factor
20	CCND1	G1/S-specific cyclin-D1

Table S4. Top 35 active ingredients in CLYF.

Rank	NO. ID	Chemical name of active ingredient
1	SQ7	Quercetin
2	CL5	20-hydroxyecdysone
3	CF3	Ginsenoside Rh2
4	DSL4	Blestriarene A
5	DSL5	Pleionesin C
6	JTS1	Psammosilenin A
7	XYS18	Phlegmariuine-N
8	ZJ3	19,20-epoxycytochalasin D
9	ZJ6	Jiangxienone
10	ZJ8	3,5,9-trihydroxyergosta-7,22-dien-6-one
11	ZJ11	Epoxycytochalasin D
12	TM12	Sucrose
13	QYS9	Arjunolic acid
14	QYS8	Glycosmisic acid
15	CL2	Diosgenin
16	ZZS1	4'-hydroxywogonin
17	ZJ1	Cytochalasin D
18	ZJ10	Cytochalasin C
19	CL3	Pennogenin
20	DSL6	Shanciol H
21	ZJ4	Trichothecin
22	TM5	M-hydroxybenzoic Acid
23	CF2	beta-sitosterol
24	CBM9	Verticinone
25	CF1	Liquiritigenin
26	SQ13	Sandaracopimarinol
27	CF10	gamma-sitosterol
28	CBM16	Solanidine
29	XYS24	Pulegone
30	ZJ12	Cerevisterol
31	QYS3	Baishouwubenzophenone
32	GC12	2-methyl-1,3,6-trihydroxyanthraquinone
33	GC16	Glycyrrhetol
34	GC37	3-methyl-6,7,8-trihydropyrrolo[1,2-A] pyrimidin-2-one
35	ZJ2	Rosenonolactone

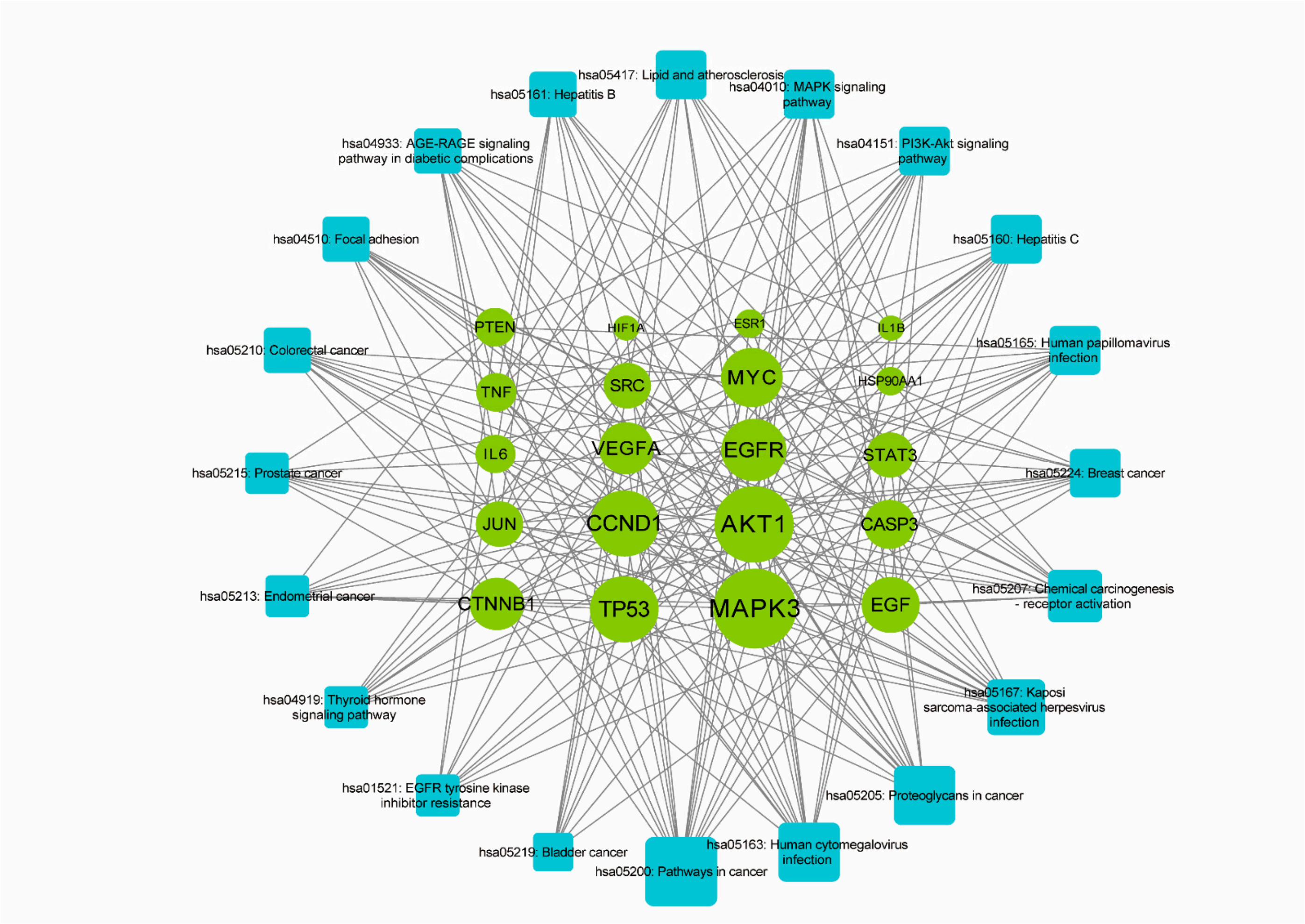

Figure S2. Interaction network of the top 20 targets of CLYF. The green nodes represent the top 20 hub genes; the blue nodes represent the pathways related to the hub targets.

Table S5. Molecular docking energy of EGFR (PDB ID 1XKK).

NO.	NO. ID	Chemical name of active ingredient	Binging energy (kcal/mol)
1	SQ7	Quercetin	−4.32
2	CL5	20-hydroxyecdysone	−3.18
3	CF3	Ginsenoside Rh2	−1.52
4	DSL4	Blestriarene A	−4.52
5	DSL5	Pleionesin C	−5.40
6	XYS18	Phlegmariuine-N	−5.14
7	ZJ3	19,20-epoxycytochalasin D	−4.25
8	ZJ6	Jiangxienone	−4.02
9	ZJ8	3,5,9-trihydroxyergosta-7,22-dien-6-one	−4.66
10	ZJ11	Epoxycytochalasin D	−4.00
11	TM12	Sucrose	−0.19
12	QYS9	Arjunolic acid	−5.54
13	QYS8	Glycosmisic acid	−2.87
14	CL2	Diosgenin	−7.29
15	ZZS1	4'-hydroxywogonin	−4.33
16	ZJ1	Cytochalasin D	−4.17
17	CL3	Pennogenin	−7.24
18	DSL6	Shanciol H	−3.42
19	ZJ4	Trichothecin	−4.87
20	TM5	M-hydroxybenzoic acid	−3.40
21	CF2	beta-sitosterol	−5.78
22	CBM9	Verticinone	−6.36
23	CF1	Liquiritigenin	−4.50
24	SQ13	Sandaracopimarinol	−5.57
25	CF10	gamma-sitosterol	−5.23
26	CBM16	Solanidine	−7.65
27	XYS24	Pulegone	−4.46
28	ZJ12	Cerevisterol	−4.92
29	QYS3	Baishouwubenzophenone	−2.61
30	GC12	2-methyl-1,3,6-trihydroxyanthraquinone	−5.23
31	GC16	Glycyrrhetol	−5.78
32	ZJ2	Rosenonolactone	−7.07

Table S6. Molecular docking energy of TP53 (PDB ID 8DC6).

NO.	NO. ID	Chemical name of active ingredient	Binging energy (kcal/mol)
1	SQ7	Quercetin	−1.95
2	CL5	20-hydroxyecdysone	−1.13
3	CF3	Ginsenoside Rh2	−1.24
4	DSL4	Blestriarene A	−0.92
5	DSL5	Pleionesin C	−0.85
6	XYS18	Phlegmariuine-N	−2.11
7	ZJ3	19,20-epoxycytochalasin D	−2.53
8	ZJ6	Jiangxienone	−1.47
9	ZJ8	3,5,9-trihydroxyergosta-7,22-dien-6-one	−1.67
10	ZJ11	Epoxycytochalasin D	−2.84
11	TM12	Sucrose	1.46
12	QYS9	Arjunolic acid	−2.79
13	QYS8	Glycosmisic acid	−0.22
14	CL2	Diosgenin	−4.60
15	ZZS1	4'-hydroxywogonin	−1.69
16	ZJ1	Cytochalasin D	−2.05
17	CL3	Pennogenin	−3.34
18	DSL6	Shanciol H	−1.13
19	ZJ4	Trichothecin	−2.44
20	TM5	M-hydroxybenzoic acid	−1.47
21	CF2	beta-sitosterol	−3.32
22	CBM9	Verticinone	−4.01
23	CF1	Liquiritigenin	−2.56
24	SQ13	Sandaracopimarinol	−3.51
25	CF10	gamma-sitosterol	−2.45
26	CBM16	Solanidine	−5.03
27	XYS24	Pulegone	−3.03
28	ZJ12	Cerevisterol	−2.30
29	QYS3	Baishouwubenzophenone	−1.4
30	GC12	2-Methyl-1,3,6-trihydroxyanthraquinone	−1.73
31	GC16	Glycyrrhetol	−4.07
32	ZJ2	Rosenonolactone	−3.60

Table S7. Molecular docking energy of AKT1 (PDB ID 6NPZ).

NO.	NO. ID	Chemical name of active ingredient	Binging energy (kcal/mol)
1	SQ7	Quercetin	−1.93
2	CL5	20-Hydroxyecdysone	−1.31
3	CF3	Ginsenoside Rh2	−1.15
4	DSL4	Blestriarene A	−1.64
5	DSL5	Pleionesin C	−1.10
6	XYS18	Phlegmariuine-N	−3.37
7	ZJ3	19,20-epoxycytochalasin D	−2.29
8	ZJ6	Jiangxienone	−0.55
9	ZJ8	3,5,9-trihydroxyergosta-7,22-dien-6-one	−1.51
10	ZJ11	Epoxycytochalasin D	−2.13
11	TM12	Sucrose	1.34
12	QYS9	Arjunolic acid	−3.19
13	QYS8	Glycosmisic acid	−0.55
14	CL2	Diosgenin	−3.09
15	ZZS1	4'-hydroxywogonin	−2.20
16	ZJ1	Cytochalasin D	−3.66
17	CL3	Pennogenin	−3.27
18	DSL6	Shanciol H	−0.93
19	ZJ4	Trichothecin	−2.94
20	TM5	M-hydroxybenzoic acid	−2.30
21	CF2	beta-sitosterol	−2.22
22	CBM9	Verticinone	−3.92
23	CF1	Liquiritigenin	−3.35
24	SQ13	Sandaracopimarinol	−3.17
25	CF10	gamma-sitosterol	−1.83
26	CBM16	Solanidine	−4.28
27	XYS24	Pulegone	−3.39
28	ZJ12	Cerevisterol	−1.36
29	QYS3	Baishouwubenzophenone	−1.74
30	GC12	2-methyl-1,3,6-trihydroxyanthraquinone	−2.65
31	GC16	Glycyrrhetol	−3.41
32	ZJ2	Rosenonolactone	−3.97

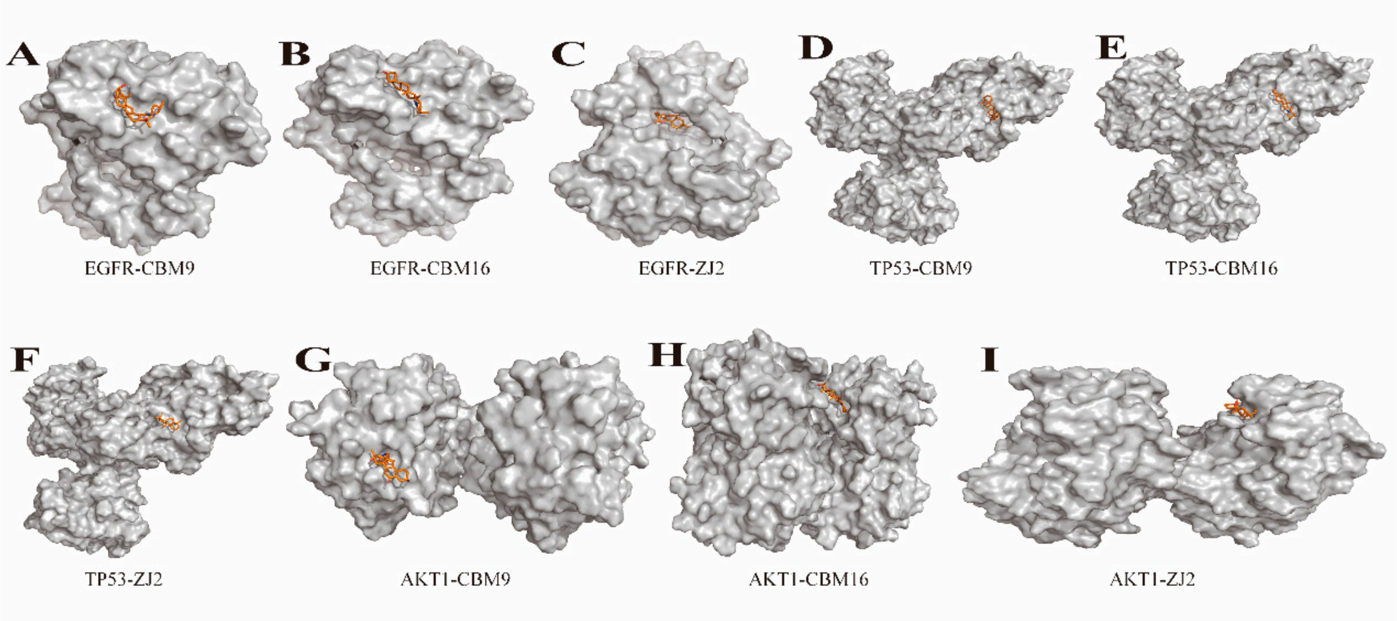

Figure S3. Three-dimensional visualization of molecular docking between the core therapeutic targets and the corresponding active compound of CLYF. (A) EGFR and CBM9. (B) EGFR and CBM16. (C) EGFR and ZJ2. (D) TP53 and CBM9. (E) TP53 and CBM16. (F) TP53 and ZJ2. (G) AKT1 and CBM9. (H) AKT1 and CBM16. (I) AKT1 and ZJ2.

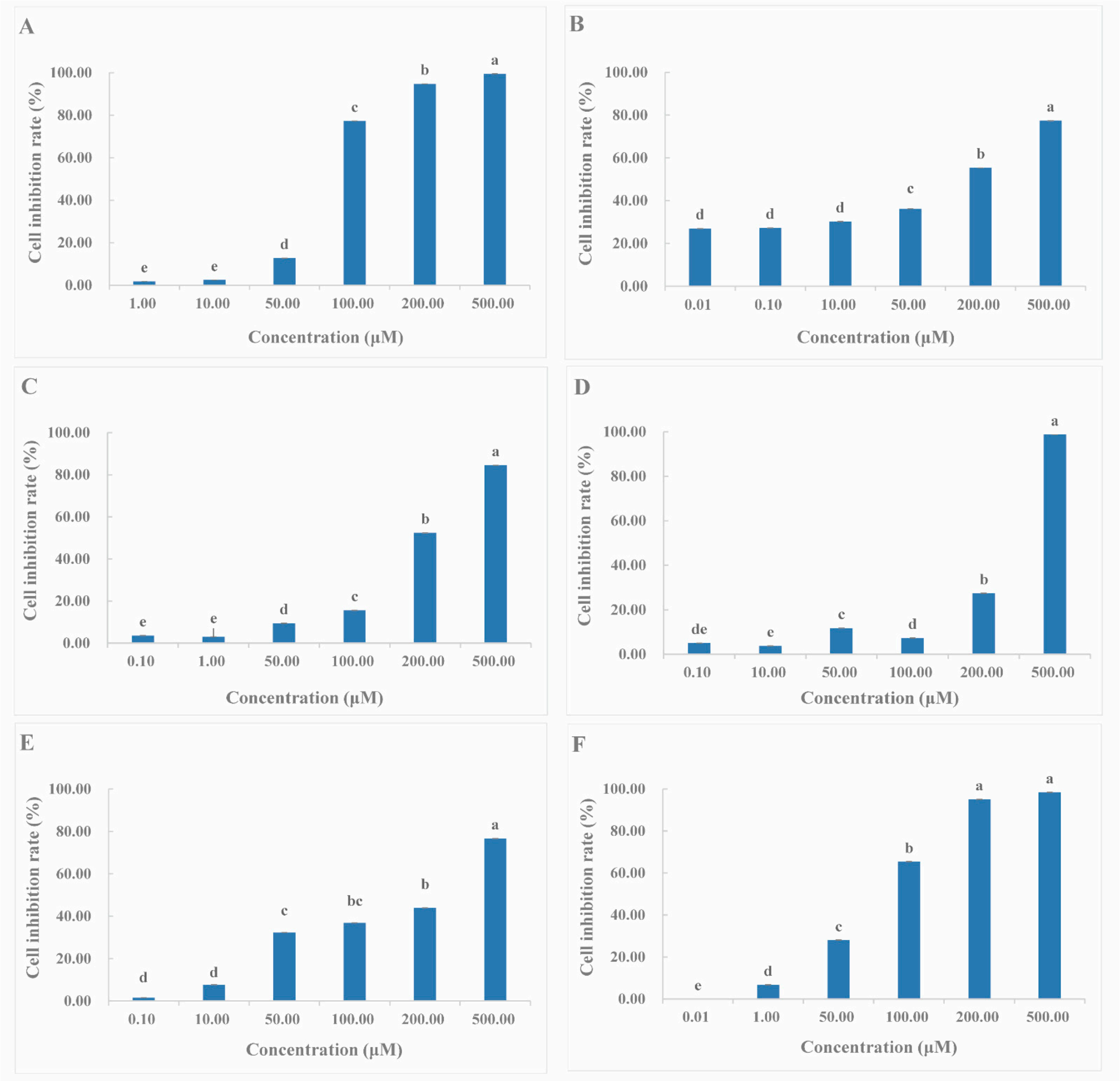

Figure S4. Inhibitory effect of CBM9 (A), CBM 16 (B), SQ7 (C), CL2 (D), CL3 (E), and CF3 (F) on HepG2 cells. Different lowercase letters indicate significant differences ($P<0.05$).

Abbreviations

TCM, traditional Chinese medicine.

Table S8. Statistical analysis of relative expression level of the core targets EGFR, TP53, and AKT1 in HepG2 cells after treatment with CLYF-A.

		**EGFR**	**TP53**	**AKT1**
Control group	DMSO	0.53 ± 0.03	0.24 ± 0.03	0.58 ± 0.01
Drug group	CLYF-A	0.21 ± 0.02*	0.82 ± 0.01*	0.25 ±0.02*

Note: Data are presented as means ± SDs from three repeated experiments. These data are statistically processed, and "*" indicates a significant difference between the CLYF-A group and the control group (*P*<0.05). This table represents the content of Figure 14 in the article.

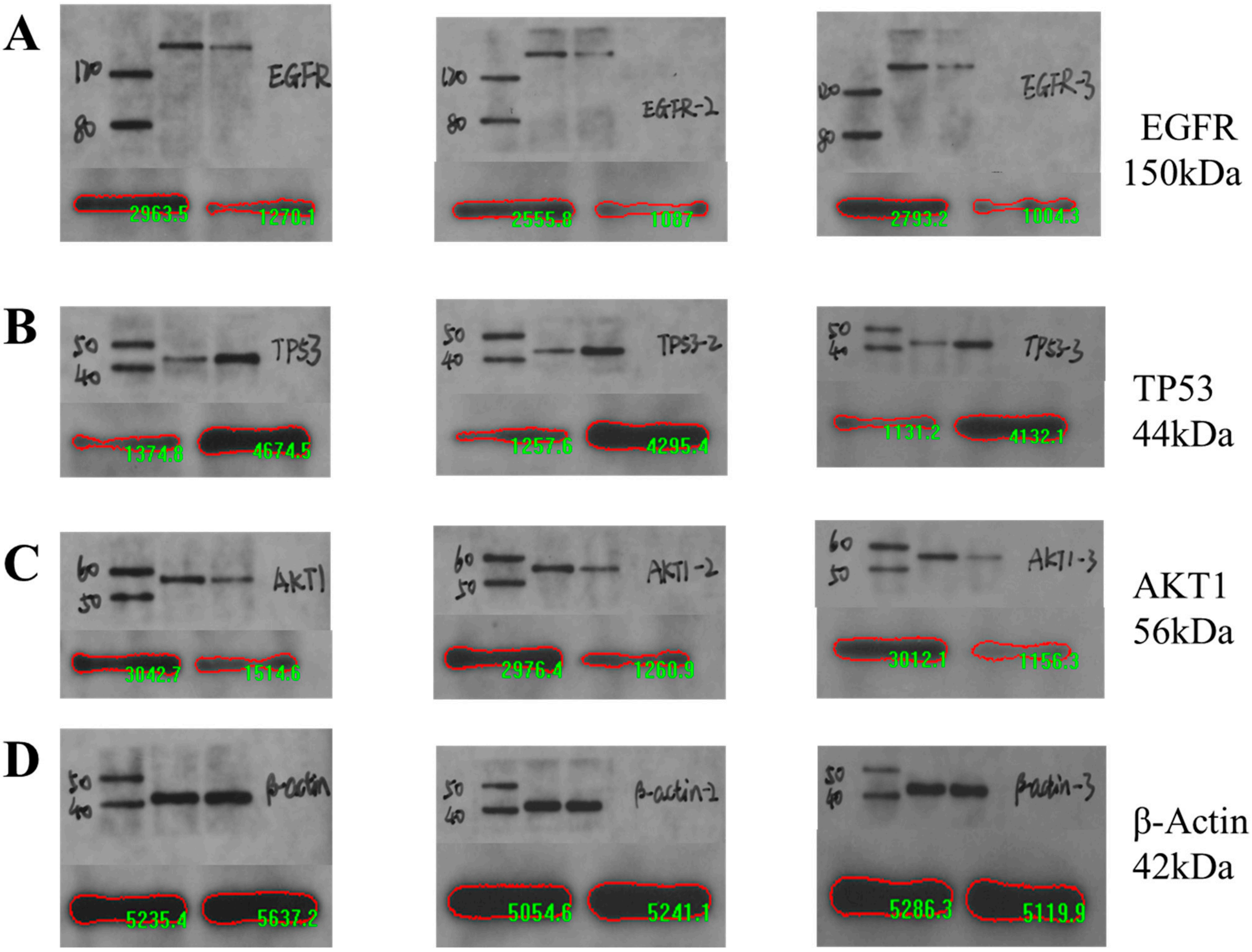

Figure S5. Figures for the original data of the Western blot experiments repeated three times independently in the article. (A) EGFR. (B) TP53. (C) AKT1. (D) β-Actin.
